# Modeling Phase Separation in Polymer-Derived Silicon Carbonitride Ceramics through Extended Machine Learning Molecular Dynamics

**DOI:** 10.1021/acsnano.6c11958

**Published:** 2026-10-06

**Authors:** Fabien Mortier, Sylvian Cadars, Olivier Masson, Mauro Boero, Guido Ori, Yun Wang, Samuel Bernard, Assil Bouzid

**Affiliations:** † Institut de Recherche sur les Céramiques, UMR 7315 CNRS-Université de Limoges, Centre Européen de la Céramique, 12 rue Atlantis, 87068 Limoges, France; ‡ University of Strasbourg, CNRS, ICube Laboratory UMR 7357, F-67412 Illkirch, France; § ADYNMAT CNRS consortium, F-67034 Strasbourg, France; ∥ Université de Strasbourg, CNRS, Institut de Physique et Chimie de des Matériaux de Strasbourg, UMR 7504, F-67034 Strasbourg, France; ⊥ School of Environment and Science, Griffith University, Gold Coast 4222, Australia

**Keywords:** ceramics, carbon, atomic-scale, phase separation, amorphous, polymer-derived, machine learning potentials, nanoscale

## Abstract

Polymer-derived ceramics combine the thermal stability of ceramics with the versatile properties of carbon domains, but modeling their atomic-scale evolution during processing remains elusive because of the limitations of traditional computational methods. To address this issue, here, we develop and apply a machine learning interatomic potential for hydrogen-containing silicon carbonitride-based (SiCNH) ceramics, trained on a diversified database of over 9000 configurations—including amorphous models, high-temperature states, surfaces, and crystal structure predictions—to capture the full complexity of these materials. This potential enables large-scale molecular dynamics simulations of 8000-atom systems, revealing the atomic-scale evolution of the polymer-derived ceramic during thermal treatment. A key result of this work is the occurrence of a phase separation, where carbon domains progressively nucleate from the amorphous SiCN matrix during thermal processing, forming distinct graphene-like sheets while preserving the integrity of the ceramic network. The resulting models reproduce the experimental atomic pair distribution functions, supporting our approach and providing microscopic explanations for the material combination of the ceramic and graphitic properties. In this process, defective 5- and/or 7-member carbon rings mediate the transformation to stable 6-member aromatic structures. These findings provide atomic-scale information about the thermal stability and structural transformation pathways of polymer-derived ceramics and demonstrate the applicability of the methodology to complex amorphous systems at experimentally relevant scales.

## Graphical abstract

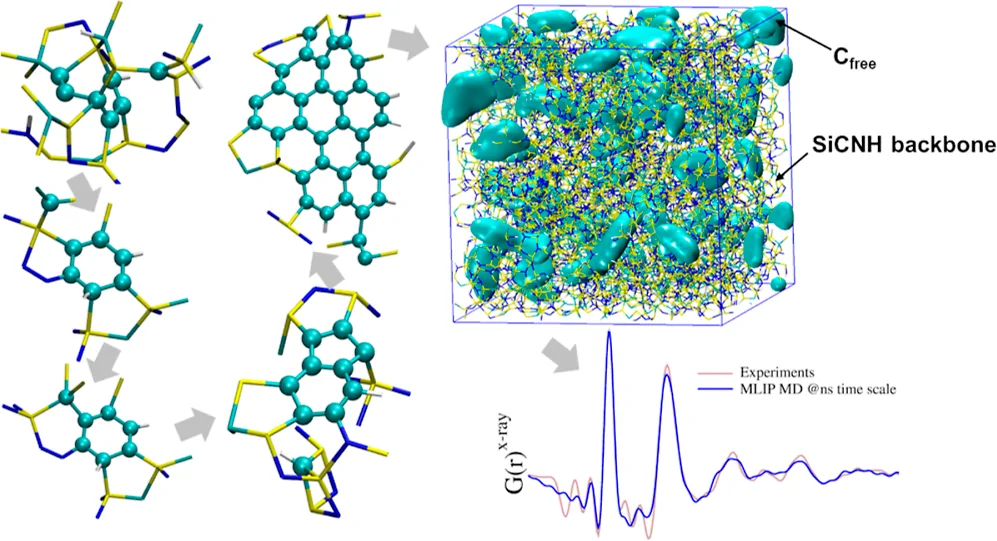

## 1 Introduction

Polymer-derived ceramics (PDCs) are advanced materials synthesized through pyrolysis of preceramic polymers such as polysilazanes, inorganic polysiloxanes, or polycarbosilanes.^1,2^ This synthetic route, first pioneered by Fritz and Raabe in the 1950s and later expanded by Ainger, Herbert, and Chantrell in the 1960s, enables the production of ceramics with tailored compositions and microstructures at relatively low temperatures (800–1500 °C) compared to conventional ceramic manufacturing.^3–5^ PDCs can adopt complex shapes and are compatible with several shaping techniques such as additive manufacturing, fiber spinning, or surface coating. In addition, thanks to their chemical and structural diversity, their thermal, electrical, and mechanical properties can be tuned.^1,6–8^ Among the most studied PDC systems, one can cite silicon carbide (SiC), silicon nitride (Si_3_N_4_), silicon oxycarbide (SiOC), and silicon carbonitride (SiCN). These materials exhibit distinct microstructures and functional properties that are tightly linked to the synthesis procedure.^9,10^

Among the PDCs, SiCN materials, when derived from polysilazane polymers pyrolyzed at temperatures below 1400 °C, feature a particularly interesting complex microstructure that arises from the nanoscale phase separation between an amorphous silicon-based network, typically composed of SiC_*x*_N_4–*x*_ tetrahedra, and a “free-carbon” phase with a degree of organization that depends on the pyrolysis temperature.^11–13^ This phase separation is driven by the decomposition of organic groups during pyrolysis, leading to the precipitation of carbon-rich regions within the ceramic network. At temperatures below 1000 °C, the free-carbon phase consists of small, disordered sp^2^-hybridized C clusters, while at higher temperatures (above 1200 °C), it progressively organizes into a turbostratic phase consisting of more-or-less extended stacked arrangements of potentially-defected graphitic layers, with various degrees of ordering up to graphite.^12,14^ Its presence is critical for boosting the overall electrical conductivity of the material, as sp^2^-hybridized carbon domains exhibit metallic-like behavior, whereas the SiCN matrix remains semiconducting.^15,16^ Moreover, this free-carbon phase acts as a diffusion barrier, and controlling its extent and degree of organization can prevent the crystallization of Si_3_N_4_ or SiC domains, thereby improving the thermal stability of the final material.^17–20^

The atomic-scale mechanisms governing the formation and growth of the free-carbon phase within the SiCN matrix remain poorly understood. Experimental techniques such as transmission electron microscopy (TEM), Raman spectroscopy, and solid-state NMR provide complementary information about the structure of SiCN ceramics and their free-carbon phase. TEM can reveal graphitic domains but does not provide direct information on how these domains nucleate and grow at the atomic scale.^12^ Raman spectroscopy gives access to the presence and degree of organization of sp^2^-hybridized carbon,^14^ while solid-state NMR provides detailed information on the local environments of Si and C atoms.^21^ However, these techniques do not directly resolve the sequence of atomic rearrangements governing the carbon nucleation, growth, and phase separation. Atomistic simulations therefore provide a complementary approach to investigating these microscopic mechanisms and connecting the experimentally observed structural features to their underlying atomic-scale evolution.

Early computational studies of PDCs relied on empirical potentials, such as the Tersoff potential, or reverse Monte Carlo (RMC) methods. Dürr et al.^22^ used RMC to model a Si_24_C_43_N_33_ composition, confirming phase separation between Si_3_N_4_-like tetrahedra and amorphous carbon. Matsunaga et al.^23^ and Liao et al.^24^ employed Tersoff potentials to study diffusion and mechanical properties, but these models lacked chemical reactivity and turned out to be insufficient to capture mixed SiC_*x*_N_4–*x*_ environments. As shown by Kroll, first-principles molecular dynamics (FPMD) on Si_40_C_40_N_40_ systems demonstrated the occurrence of phase separation and the formation of carbon clusters, but the small system sizes (120 atoms) and short simulation times (40 ps) limited insights into the long-term structural evolution.^25^ In another work, Amkreutz et al.^26^ used density functional-based tight binding (DFTB) to study the phase separation in Si_37_C_32_N_31_ systems, proposing that the free-carbon phase could exist as either small clusters or extended layered structures. While insightful, these studies left several open questions about the dynamical evolution of the free-carbon phase.

In a recent FPMD-based study,^27^ we addressed some of these limitations by focusing on a hydrogen-containing silicon carbonitride (SiCNH) PDC of composition Si_32_C_25_N_24_H_19_ derived from $-{\left[Si\left({CH}_{3}\right)\left(CH={CH}_{2}\right)-NH-\right]}_{0.2}-{\left[Si\left(H\right)\left({CH}_{3}\right)-NH-\right]}_{0.8}-$ polyvinylsilazane. This Si–C–N–H composition is retained as the reference system in the present work because it has been experimentally characterized and previously investigated by FPMD, providing a consistent basis for extending the simulations to larger spatial and temporal scales. Using the ceramicNetworkBuilder algorithm (available in the Python Atomic Modeling Analyzes library^28^), we generated several initial configurations differing in the organization and networking of the free-carbon phase, including clusters, chains, and pre-inserted graphitic layers. After thermal annealing, the models were validated against experimental pair distribution functions (PDFs), revealing that models where carbon is essentially made of poorly spatially correlated layers, rather than extended and stacked layers, best match the experimental data. While this work provided an atomic-scale validation of plausible free-carbon phase structures and atomic local environment within PDCs, it is still constrained by the small system sizes (400 atoms) and short time scales (hundreds of picoseconds) accessible to FPMD. As such, it did not capture the medium-/long-range dynamics of carbon domain growth or the full complexity of phase separation over experimentally relevant time and/or size scales.

The breakthrough of machine-learning interatomic potentials (MLIPs) has revolutionized modeling of complex materials by combining the accuracy of ab initio methods with the efficiency of classical force fields. As far as PDC materials are concerned, Kroll et al.^29,30^ recently introduced machine-learning interatomic potentials for Si–C–N–H and Si–C–O–H systems based on moment tensor potentials (MTPs). In these studies, the reported final training sets comprise 3417 structures for Si–C–O–H^29^ and 2104 structures for Si–C–N–H.^30^ While both articles describe the principal sources of configurations (e.g., molecules, polymers, crystalline and amorphous structures, and high-temperature trajectories), the detailed sampling distribution across compositions, thermodynamic conditions, and reactive motifs is not fully specified. In addition, the increased chemical complexity of Si–C–N–H required the use of a higher-order MTP in ref 30, while a level-12 MTP was initially targeted, as previously used for Si–C–O–H,^29^ a level-18 MTP was ultimately required to adequately describe the diverse nitrogen-bonding environments, including reactive NH_3_ and the strongly bonded N_2_ molecule. This increase in the MTP level resulted in an approximately fivefold increase in computational cost compared with the level-12 potential, illustrating the accuracy-efficiency trade-off associated with increasing the complexity of the MTP representation.

Despite these limitations, Kroll et al. demonstrated that MTP-based MLIPs can reproduce key near-equilibrium properties and enable the simulation of polymer-to-ceramic conversion at size and time scales inaccessible to ab initio methods. For Si–C–O–H, the MTP-MLIP reproduced vibrational signatures and captured the main features of polysiloxane pyrolysis, including the emergence of mixed Si-centered tetrahedra and the precipitation of graphitic carbon in C-rich compositions, with simulations reaching million-atom models over nanoseconds.^29^ For Si–C–N–H systems, the MTP-MLIP enabled pyrolysis simulations of polysilazanes to amorphous SiCNH with plausible carbon segregation morphologies and supported kinetic analysis from simulated thermogravimetric data.^30^ Nevertheless, as these potentials were primarily developed to enable reactive simulations of polymer pyrolysis, the nucleation and growth of the free-carbon phase as well as its interaction with the surrounding SiCO/SiCN matrix were not systematically investigated as central objectives of those works. Furthermore, a direct quantitative comparison of the predicted carbon-phase morphology within the SiCN matrix to experiment remains limited. This makes it difficult to assess the reliability of the MLIPs specifically for describing the final structure of the phase-separated ceramic.

In the present study, we overcome these limitations by developing and applying an accurate MLIP for Si–C–N–H systems, trained on a large database containing over 9000 configurations featuring a high degree of structural and compositional diversity to ensure model robustness. This database includes various amorphous bulk Si–C–N–H models, high-temperature overheated states, surfaces and interface models to describe free-carbon domain boundaries, crystal structure prediction models to sample rare but potentially critical bonding environments, and amorphous carbon and silicon structures to ensure transferability across extreme compositions. Our MLIP, built using the MACE framework,^31,32^ achieves root mean square errors of 12.4 meV/atom for energies and 149 meV/Å for forces on an unseen test database. This enables large-scale molecular dynamics simulations of 8000-atom systems over nanosecond time scales, providing insights into the atomic-scale mechanisms driving carbon domain nucleation and growth in amorphous Si–C–N–H systems. Overall, we establish a framework for the study of complex phenomena in amorphous ceramics, relying on a structurally diversified database together with equivariant machine-learning interatomic potentials.

## 2 Results and Discussion

### 2.1 MLIP Validation

This manuscript describes analyses of the atomic-to-nanometer-scale structure and organization mechanisms of Si–C–N–H PDCs, whose reliability is profoundly conditioned by the quality of the underlying atomic-scale modeling engine, and specifically the MLIP model (see Section 4.2) trained from a methodically constructed database of structures and associated DFT calculations (Section 4.1). Here, we first evaluate the accuracy of the MLIP model, before proceeding, in Section 2.2, to the evaluation of structural models produced with MLIP-MD simulations (Section 4.3), as prerequisites, in our opinion, to the subsequent exploration of the carbon-phase structuration that is the main focus of this manuscript.

Figure 1 shows the comparison between final MLIP predictions and DFT reference values for energies and forces across all configuration classes (detailed in Sections 4.1.1–4.1.3) in the (previously unseen) test set. The performance of the potential is quantified using root mean square errors (RMSEs) and relative RMSE (RRMSE) for each configuration class (see Table 1). The overall RMSEs of the model are 12.4 meV/atom for energies and 149 meV/Å for forces, with a RRMSE of 10.2% for forces. By looking at configuration classes individually, we observe that RMSEs on energies fall below 10 meV/atom, and RMSEs on forces are of the order of 100 meV/Å for SiCNH-periodic, SiCNH-surface, Amorphous-Carbon-200, Amorphous-Carbon-250, and Amorphous-Silicon. For SiCNH-periodic-4000K, while RMSE on energy is also below 10 meV/atom, the value on forces reaches 218 meV/Å. This rise in force RMSE comes from the higher energy of the configurations produced at high temperature (>2000 K) included in this class. This higher absolute RMSE is nevertheless not followed by a significant increase of the RRMSE, which is of the same order than the RRMSE of the other classes of configuration (between 8 and 13%). The absolute RMSEs on both energies and forces are relatively higher for the Crystal-Structure-Prediction configurations than for other classes (due to the higher energies and forces of these configurations). However, its RRMSE on forces is only of 9.2%. These performances are particularly noteworthy because crystalline configurations of the Crystal-Structure-Prediction class contain many high-energy atomic environments far from the typical amorphous structures encountered in FPMD simulations. They highlight the accuracy and transferability of the potential, making it suitable for large-scale molecular dynamics simulations of Si–C–N–H systems. In the following sections, we apply this potential to study the atomic-scale mechanisms governing carbon nucleation and growth in SiCNH PDCs.

**Figure 1:**
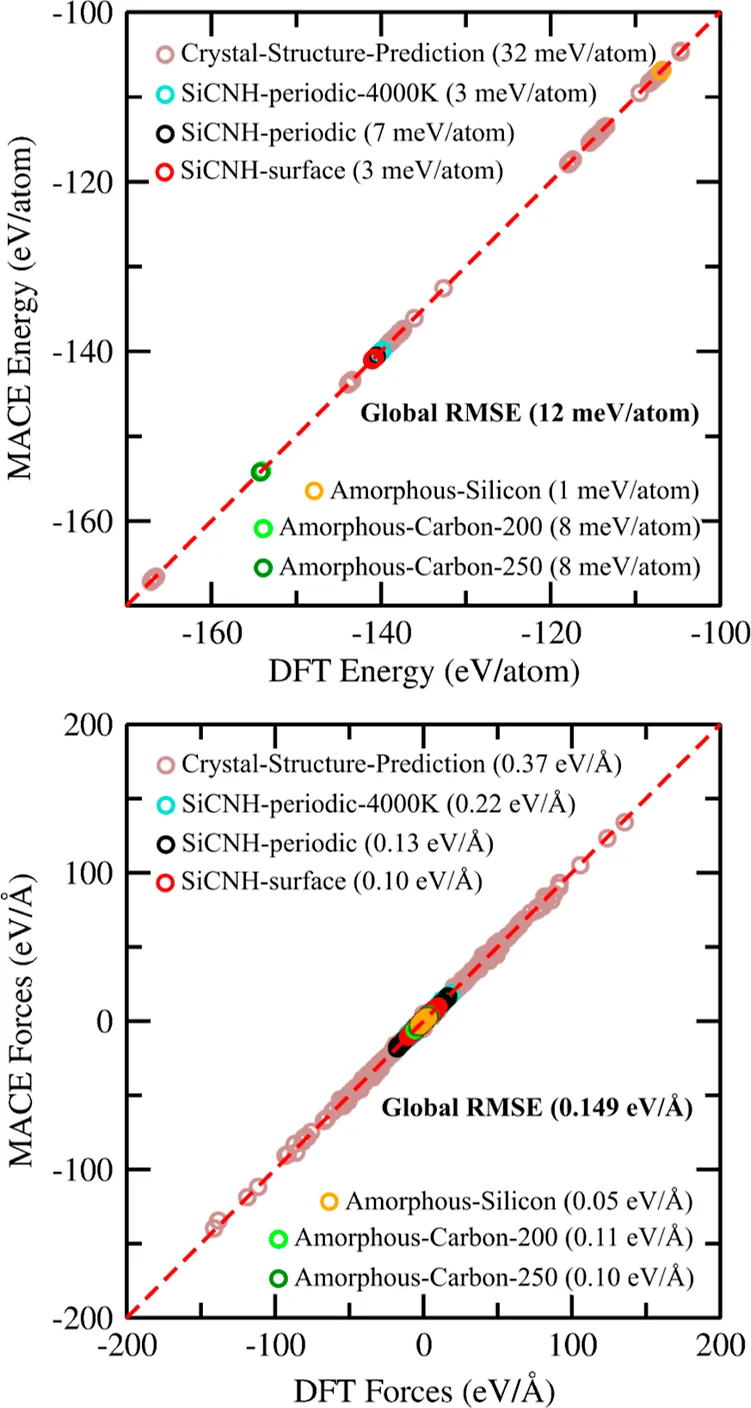
Comparison of MLIP-predicted energies and forces with DFT reference values for the test set. Root mean square errors are given between parentheses.

**Table 1:** RMSE and RRMSE Values for the MLIP on the Test Set, Compared to DFT Reference Calculations

class	RMSE energy	RMSE forces	RRMSE forces
	(meV/atom)	(meV/Å)	(%)
SiCNH-periodic	7.1	128	9.5
SiCNH-surface	3.0	104	8.5
SiCNH-periodic-4000K	3.3	218	12.6
amorphous-carbon-200	7.9	110	12.0
amorphous-carbon-250	7.8	103	10.9
amorphous-silicon	1.1	51.3	9.1
crystal-structure-prediction	31.8	373	9.2
total	12.4	149	10.2

To further assess the dynamical accuracy of the MLIP beyond energy and force benchmarks, we directly compared the vibrational density of states (VDOS) obtained from FPMD and MLIP-MD simulations on the same 200-atom Si–C–N–H model from ref 27 subjected to comparable thermal annealing protocols. The total and element-resolved VDOS obtained from the final 20 ps trajectories at 300 K show very good agreement between FPMD and MLIP, reproducing the same main vibrational bands and spectral shapes for Si, C, N, and H. The calculated frequency domains are also consistent with experimental vibrational signatures reported for related polysilazane-derived materials^33,34^ and with previous DFT vibrational assignments for hydrogenated silicon carbonitride systems.^35^ Details of the protocol and comparison are provided in Section S6 and Figure S5 of the Supporting Information

### 2.2 Validation of the SiCNH Models

Using the validated MLIP, we performed molecular dynamics simulations of four 8000-atom SiCNH models with the composition Si_32_C_25_N_24_H_19_. The models differ in their initial carbon organization and thermal treatment: SiCNH8000-Random starts from an unconstrained random atomic distribution, SiCNH8000-CNB from an amorphous network constructed based on local atomic bonding and coordination constraints, and SiCNH8000-CL from a network containing pre-formed carbon sheets. An additional model, SiCNH8000-CNB_2200, was obtained by extending the thermal treatment of SiCNH8000-CNB to 2200 K (see Figure 2a). Details of the model construction and molecular dynamics protocols are provided in the Methods section.

**Figure 2:**
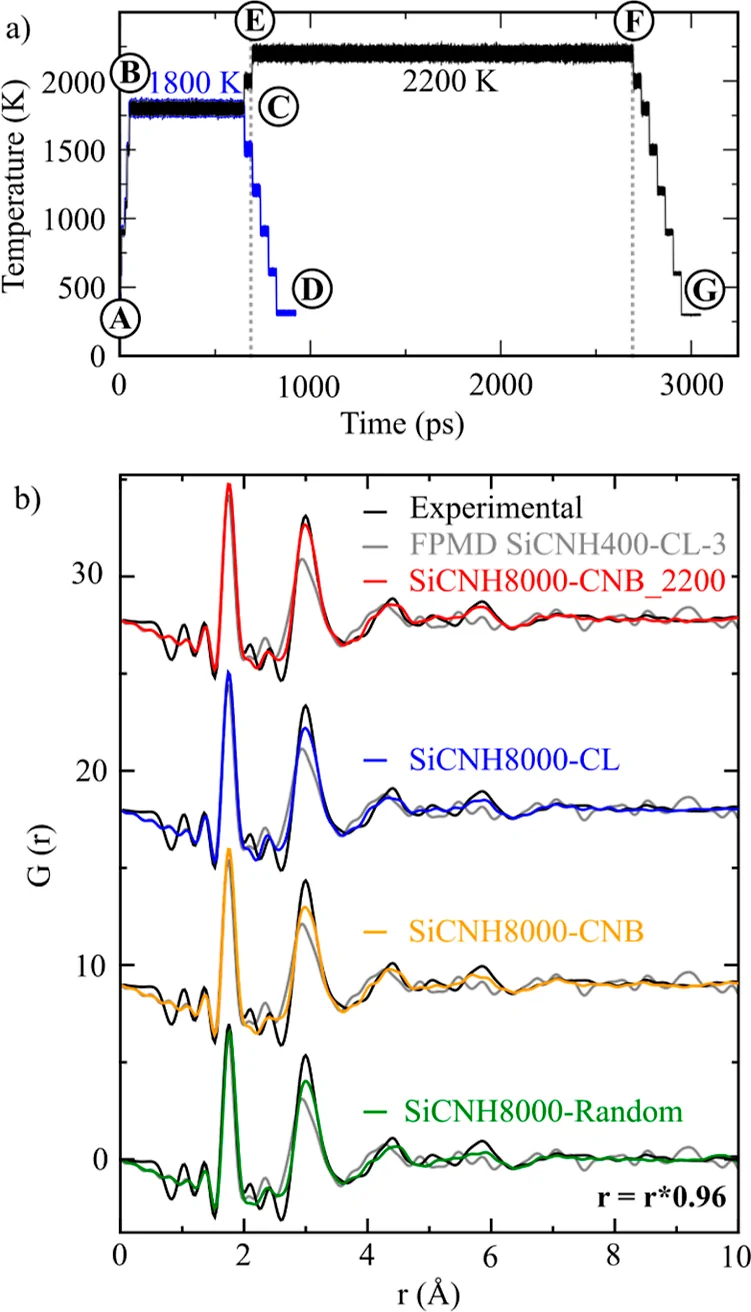
(a) Thermal cycles applied to the 8000-atom models using the MLIP. The extended high-temperature thermal cycle shown in black was used to obtain the SiCNH8000-CNB_2200 model, while other models were obtained with the shorter thermal cycle shown in blue. (b) X-ray scattering total pair distribution functions (*G*(*r*)) computed from the 8000-atom models compared with the experimental *G*(*r*) reported in ref 27 and with the FPMD SiCNH400-CL-3 model from the same reference. Simulated data use *r* values scaled by 0.96 for optimal alignment with experimental peaks (see text).

Figure 2b presents a comparative analysis of the total PDFs of our four 8000-atom models and the experimental X-ray scattering total pair distribution function *G*(*r*) of the SiCNH ceramic reported in ref 27, where the complete experimental procedure and data treatment are described.

As discussed in ref 27, the FPMD SiCNH amorphous model PDFs show a better agreement with the experimental PDF (especially for peak positions) when applying an *r* scaling factor slightly lower than 1. This scaling factor was explained mainly by the adopted DFT setup, which tends to slightly overestimate atomic bond lengths. The resulting r-scaled model density was 11% larger than the experimental one, which was attributed to an underestimation of the measured density (by helium pycnometry) due to the presence of a large fraction of closed porosity in the sample.^27^ Given that our MLIP was trained on the FPMD data, the same trends are also observed in the MLIP-generated models studied in this work. Indeed, the best agreements with experimental data are obtained by applying similar r-scaling factors of approximately 0.96 (see Figure 2b) leading to a model density 13% larger compared to the experimental value. We note that no independent NPT equilibrium density was determined in the present work as the simulations were intentionally performed at the experimental density for direct structural comparison. The *r*-scaling discussed above therefore provides an indirect estimate of the density and bond-length bias inherited from the exchange functional (PBE) and van der Waals corrections (D3 type).

The PDFs simulated from all four MLIP-MD models reveal a considerably increased agreement with experimental data, compared with the 400-atom FPMD model, regardless of their different initial configurations. Focusing on peak intensities, the two experimental PDF peaks centered at 1.37 Å and 1.76 Å are well reproduced by all our MLIP-MD models. These peaks correspond, respectively, to C–C bonds and Si–C and Si–N bonds, as previously identified.^27,36^ In addition, the C–(C)–C peak at 2.42 Å, which in our previous FPMD models (particularly those with large stacked carbon sheets) was often overestimated, now shows a better match with the experimental PDFs, suggesting an improved description of the atomic-scale structure as model size and simulation times are increased. Small discrepancies are nevertheless observed, such as the one around 2 Å, where the low-intensity experimental peak is not fully reproduced by either the MLIP-MD model or the earlier FPMD models. This is likely due to imperfect data corrections to obtain the experimental PDF, which systematically produces more pronounced artifacts in the region of low *r* values. The most striking difference between the PDFs of the FPMD model and of the 8000-atom MLIP-MD models is observed for the peak centered at around 3.00 Å, which features increased intensity and slightly reduced width in the latter, in better agreement with the experiment, particularly for the SiCNH8000-CNB_2200 model. This peak encompasses multiple correlations, including N–(Si)–N, N–(Si)–C, Si–(N)–Si, Si–(C)–Si, and C–(C–C)–C. The intensity difference between calculated and experimental PDFs was previously attributed (based on FPMD models) to the presence of Si–Si bonds or –Si–X–Si–Y– 4-member rings (X, Y = N or C) disrupting the organization of the SiCN network. The increased intensity of this peak at 3.00 Å in the large MLIP-MD models therefore suggests a slight improvement in the description of the network when using the MLIP potential, which can be attributed to the large time and size scales of these simulations allowing the system to achieve a better relaxation. Finally, while the peaks located beyond 4 Å remain broad and of low intensity, indicating a limited order at these longer correlation lengths, they are better reproduced by the large models than by previous FPMD models.

Overall, the PDFs of our 8000-atom models exhibit a notably better agreement with the experimental reference compared to models obtained from FPMD simulations. Focusing on the MLIP models, SiCNH8000-CL and particularly SiCNH8000-CNB_2200 show an overall better agreement with the experimental PDF than the SiCNH8000-Random and SiCNH8000-CNB models. This is particularly visible for the large *r* values.

In order to quantitatively compare the experimental and the modeling results, we minimized the RMSE between experimental and simulated data by optimizing the *r* scaling factor. In addition, we also optimized the amplitude of the calculated PDFs, as an imperfect data normalization procedure might affect the experimental PDF intensity scaling. The obtained optimized PDFs are shown in Figure S7 in the Supporting Information and the corresponding RMSE results are summarized in Table 2. This analysis confirms the superiority of SiCNH8000-CL and particularly SiCNH8000-CNB_2200 models in reproducing the experimental PDFs.

**Table 2:** Results of Optimization of Simulated PDF Amplitude and *r* Scaling for the Best Match with Experimental Data

model	amplitude scaling	*r* scaling	RMSE
SiCNH400-CL-3 (FPMD,^27^)	1.313	0.977	0.521
SiCNH8000-random	1.290	0.958	0.311
SiCNH8000-CNB	1.233	0.961	0.315
SiCNH8000-CL	1.237	0.962	0.289
SiCNH8000-CNB_2200	1.147	0.961	0.260

### 2.3 Structural Characterization of the Amorphous Models

#### 2.3.1 Partial Pair Distribution Functions

Having established the agreement of the large-scale models with the experimental total PDF, we now examine their atomic-scale structure in greater detail. Figure 3 presents the partial PDFs for the four 8000-atom models, excluding hydrogen-containing pairs, which provide only minor contributions to the total PDF. Before examining the individual partial PDFs, we first point out that most major partial peaks not only have considerably smoother and more regular shapes than the corresponding peaks in the previous FPMD partials in ref 27 (which is primarily due to better statistics in 8000-atom models), but they are also significantly narrower. This suggests that many irregular environments present in FPMD models (obtained after a few tens of picoseconds of annealing) are eliminated by the nanosecond time scales accessible to MLIP simulations.

**Figure 3:**
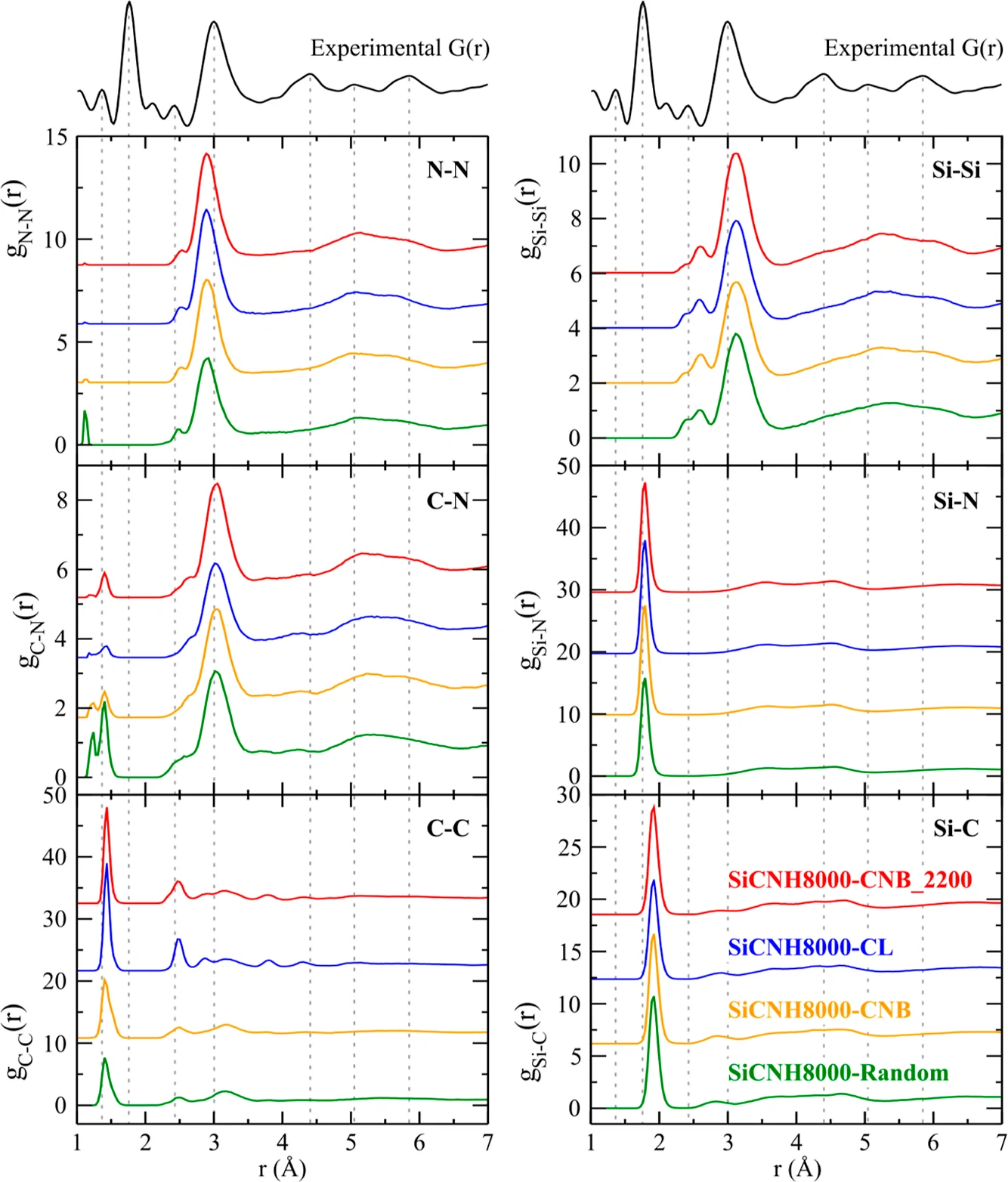
Partial pair distribution functions for the 8000-atom models.

First, the g_C–C_(*r*) functions highlight the increasing organization of carbon domains when moving from SiCNH8000-Random to SiCNH8000-CNB, SiCNH8000-CL, and finally to SiCNH8000-CNB_2200 model. The first peak centered at 1.42 Å is more intense for SiCNH8000-CL and SiCNH8000-CNB_2200 models compared to the other two, indicating a greater quantity of C–C bonds in these models. The sharpening of this peak also suggests a tendency for carbon atoms to adopt an sp^2^ configuration. Interestingly, while SiCNH8000-Random and SiCNH8000-CNB models show broad C–C correlations beyond 2 Å, SiCNH8000-CL and SiCNH8000-CNB_2200 models exhibit well-defined peaks at distances compatible with those found in typical C rings (≈ 2.47 and 2.9 Å).

The g_N–N_(*r*) functions display a peak at approximately 1.15 Å, indicating the presence of N_2_ molecules. Their numbers are 28, 2, 1, and 1 in the SiCNH8000-Random, SiCNH8000-CNB, SiCNH8000-CL, and SiCNH8000-CNB_2200 models, respectively. The larger amount in the random model originates from its unconstrained initialization, whereas the ceramicNetworkBuilder approach strongly limits the formation of such undesired environments by imposing chemically motivated bonding constraints. The small peak around 2.53 Å is due to four-atom –Si–N–Si–N– cycles, while the main N–N peak at 2.92 Å corresponds to N–(Si)–N correlations within the SiCN network.

Focusing on the g_C–N_(*r*) partial PDFs, all 8000-atom models show two peaks at 1.25 and 1.43 Å attributed to double C=N or triple C≡N bonds and to C–N single bonds, respectively. The SiCNH8000-Random model contains the highest amount of short C–N bonds, again due to its random initialization, whereas this bond type is prohibited in the setup of the ceramicNetworkBuilder program used to generate SiCNH8000-CNB, SiCNH8000-CL, and SiCNH8000-CNB_2200 initial configurations. Nevertheless, few C–N bonds occurs in these models.

Similar to N–N correlations, the main C–N peak around 3.05 Å characterizes the C–(Si)–N distances of the SiC_*x*_N_4–*x*_ tetrahedral network, and the shoulder at lower *r* values (around 2.6 Å) corresponds to –Si–C–Si–N four-atom cycles. In this case, the narrow and symmetrical shape of the main peak in MLIP-MD models is a strong indication that artefactual environments present after model initialization continue to be eliminated as high-temperature MLIP-MD simulations extend far beyond typical time scales accessible to FPMD.

The Si–Si partial PDFs are similar for all four MLIP models. The only main difference can be seen in the SiCNH8000-CNB_2200 model where the highest intensity peak at 3.2 Å, encompassing the Si–(N)–Si and Si– (C)–Si correlations, is more intense and slightly narrower, explaining the achieved best match with the experimental PDF. In addition, SiCNH8000-CL and SiCNH8000-CNB_2200 models show a slightly reduced intensity of the first peak at 2.43 Å corresponding to Si–Si bonds. These results establish that the model that underwent the longest thermal cycle leads to an improved connectivity of the SiCN network as well as a reduction in the number of Si–Si bonds. Experimentally, Si–Si bonds have been already suggested by Choong Kwet Yive et al.,^37^ although they are known to rearrange at high temperatures to more stable Si–C–Si bridges via methylene insertion.

Finally, the Si–N and Si–C atomic pairs exhibit quite similar distributions with a very intense peak centered at 1.81 Å and 1.93 Å, corresponding to Si–N and Si–C bonds, respectively.

Taken together, the above analysis shows that the best two models, SiCNH8000-CL and in particular SiCNH8000-CNB_2200, are consistent with the plausible presence of two separate phases: a carbon phase, containing sp^2^-like C atoms, and a SiCN network. This can be confirmed by inspecting the C atoms organization in the models. Figure 4 shows the projections of the simulation cells with only carbon atoms obtained at the end of the thermal annealing cycles. One can see that the best models, SiCNH8000-CL and SiCNH8000-CNB_2200, show unambiguously the presence of more or less developed carbon sheets scattered in the cell, which is less the case for the two other MLIP models. Focusing on the SiCNH8000-CNB_2200 model, the phase-separated carbon domains are actually embedded in the SiCN network as shown in the perspective view of Figure 4. These observations are compatible with the formation of the well-known free-carbon phase in PDC materials and demonstrate the ability of the present MLIP-based methodology, associated with an extended size and time scales, to achieve realistic structural models with phase separations.

**Figure 4:**
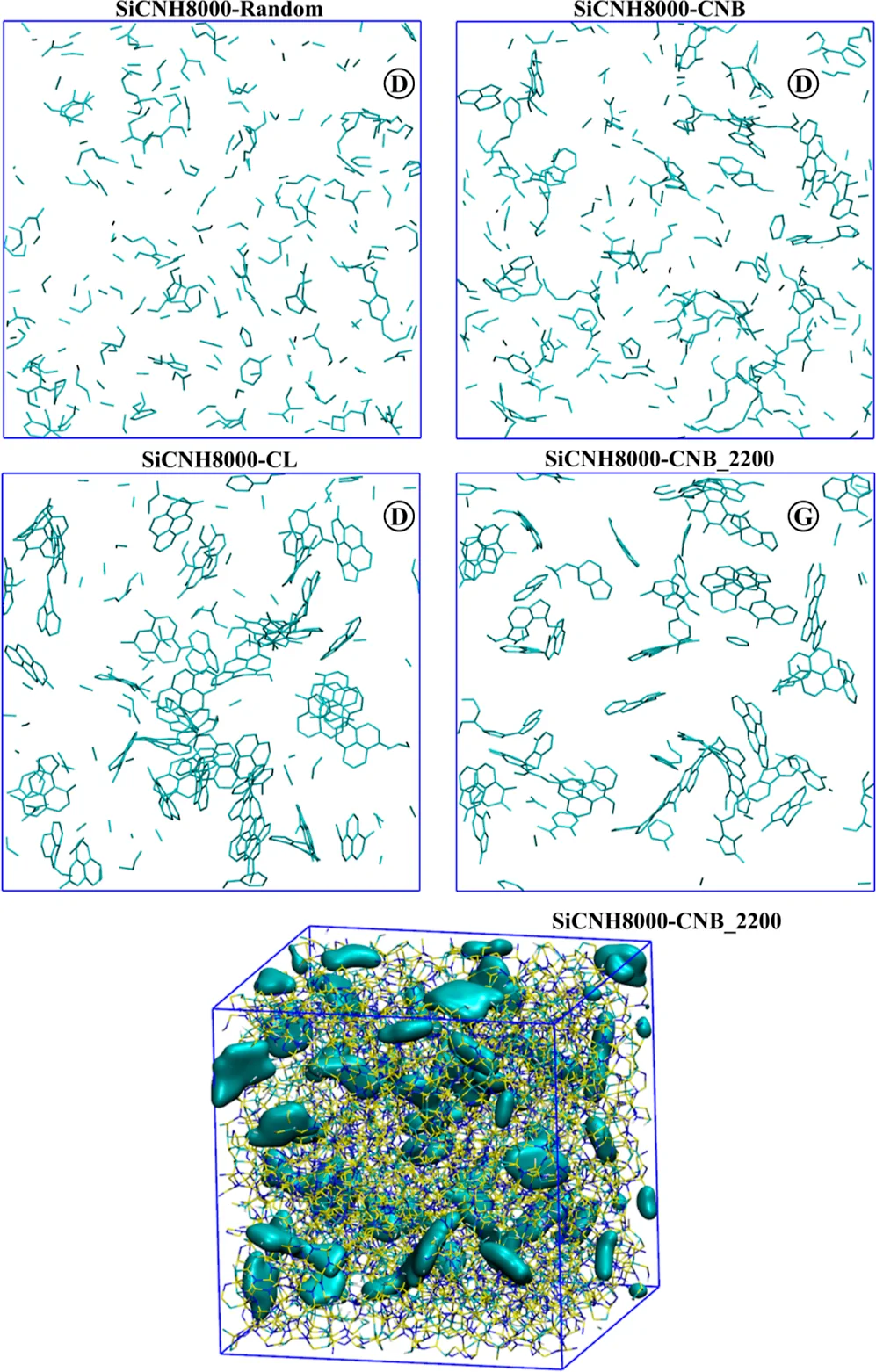
(Top) Configurations of C–C bonds in the final snapshot of models SiCNH8000-Random, SiCNH8000-CNB, SiCNH8000-CL, and SiCNH8000-CNB_2200. Capital letters refer to the position on the thermal cycles shown in Figure 2a. (Bottom) Perspective view of the final SiCNH8000-CNB_2200 model, with carbon domains highlighted in QuickSurf iso-surface representation in VMD.^38^

#### 2.3.2 Local Atomic Environments

Table 3 shows the dominant Si local environments in the four 8000-atom models, and Tables S4–S7 in the Supporting Information provide the complete list of local environments for all the models. We find that, in the four models, between 87% and 90% of silicon atoms are 4-fold coordinated, as expected from a Si-based tetrahedral network.

**Table 3:** Local Silicon Environments in the 8000-Atom Models, Showing the Fraction of Main 4-fold Coordination Motifs^a^

model	4-fold Si environments (%)				
	Si–N_4_	Si–CN_3_	Si–C_2_N_2_	Si–C_3_N	Si–C_4_
SiCNH8000-random	5.2	19.0	24.7	13.9	3.2
SiCNH8000-CNB	7.6	21.2	27.1	14.3	3.0
SiCNH8000-CL	9.5	23.3	20.5	11.4	3.1
SiCNH8000-CNB_2200	8.3	23.3	27.9	14.0	2.3

a The full tables are provided in the Supporting Information paragraph S9.

Interestingly, silicon atoms are in majority bonded to nitrogen or carbon atoms forming mixed SiC_*x*_N_4–*x*_ tetrahedra. Unmixed Si–C_4_ and Si–N_4_ represent only around 3% and less than 10% for all models, respectively. Overall, there is no significant difference between the distribution of the silicon environments in the four models. One can only note that in SiCNH8000-CL, the Si–C_2_N_2_ environments are less frequent, certainly due to the introduction of carbon sheets in the initial configuration, which reduces *de facto* the available carbon atoms that can participate in the formation of the SiCN network. Beside this, we find other less common silicon environments that involve hydrogen or other silicon atoms (see complete tables in the Supporting Information paragraph S9). Specifically, we note the existence of 3% to 4% environments such as Si–SiC_2_N and Si–SiCN_2_ in most of the models.

The analysis of the nitrogen local environments shows that it is predominantly 3-fold coordinated and essentially bonded to silicon atoms (in N–Si_3_ or N–Si_2_H environments), as expected in a SiCN network.

Table 4 shows the dominant 3- and 4-fold carbon local environments (see the full tables in the Supporting Information paragraph S9). We find that carbon atoms are primarily 4-fold (≈ 52–58%) and 3-fold (≈ 37–47%) coordinated. The 4-fold coordinated carbon atoms belong to the SiCN network, whereas the 3-fold ones essentially belong to the free-carbon domains. Focusing on the two best models, we observe that the C–C_3_ environment covers more than 15% of the carbon atoms, reflecting the occurrence of carbon layers in contrast to the SiCNH8000-Random and SiCNH8000-CNB models. Their sp^2^-like character is further supported by the geometric bond-angle analysis discussed below and in the Supporting Information The carbon atoms located at the edges of these sheets are predominantly found in 3-fold C–SiC_2_ environments, building the connection between the matrix and carbon domains. As for the C–Si_2_C environments, while their fraction was around 10% SiCNH8000-CNB model, they almost disappear after the extended annealing of the SiCNH8000-CNB_2200 model, suggesting rather limited stability of this local configuration inside the Si–C–N–H system.

**Table 4:** Fractions of the Main 3- and 4-fold Carbon Environments in the 8000-Atom Models^a^

model	3-fold C environments (%)				4-fold C environments (%)		
	C–Si_3_	C–Si_2_C	C–SiC_2_	C–C_3_	C–Si_4_	C–Si_3_H	C–Si_2_H_2_
SiCNH8000-random	4.7	7.6	6.3	3.5	21.1	22.9	6.4
SiCNH8000-CNB	3.4	10.4	12.5	6.2	17.9	21.1	6.2
SiCNH8000-CL	4.0	4.1	16.2	16.1	18.0	21.1	5.9
SiCNH8000-CNB_2200	4.9	1.0	12.3	15.0	24.2	20.8	7.5

a The full tables are provided in the Supporting Information paragraph S9.

As previously mentioned, 4-fold carbon atoms located in the matrix are primarily bonded to Si atoms and show a significant tendency to incorporate hydrogen in their neighborhood with nearly 20% of C–Si_3_H environments. Hydrogen atoms appear to be mainly bonded to carbon atoms of the matrix, playing a crucial role in stabilizing carbons that cannot form four bonds with silicon due to the constraints of the amorphous network. At the same time, the hydrogen atoms act as terminal atoms, preventing the formation of a fully continuous random SiCN network. This, in turn, can possibly provide a higher flexibility to the amorphous network. It is worth noting that only a relatively small fraction of hydrogen atoms are bonded to silicon in agreement with the lower stability of Si–H bonds in comparison to C–H ones.^39^

### 2.4 Free-Carbon Domain Characterization

The local environments found in all models are globally similar, with differences arising from the fraction of each environment. These models, due to their distinct initial configurations, may reflect different stages of material organization during experimental pyrolysis. Indeed, the SiCNH8000-CNB model represents an intermediate stage, where the amorphous network develops a few poorly organized carbon-rich domains. SiCNH8000-CNB_2200 and SiCNH8000-CL models describe a more advanced stage, characterized by the formation and stabilization of larger carbon domains within the SiCN network, where carbon atoms forming C–C bonds generally adopt sp^2^ hybridization, similar to the free-carbon phase observed experimentally.^14^ Finally, the SiCNH8000-Random model, while not representative of the early pyrolysis stages due to its nonphysical random initialization, provides insights into the nucleation of the carbon phase and particularly the construction of aromatic rings from isolated atoms. In what follows, we present the evolution of carbon domains by exploiting the four 8000-atom models.

#### 2.4.1 Evolution of C Motifs

Figure S8 in the Supporting Information shows snapshots of the carbon domains of all models at different stages of the thermal cycles. It reveals that, during the thermal annealing, carbon atoms undergo substantial organization leading to the formation of dimers, chains, and sheets. Specifically, we define five categories of carbon atoms (illustrated graphically in Figure 5e) characterizing typical structural carbon motifs and use them to track the system evolution during the thermal annealing cycle. The first category corresponds to “single” carbon atoms only bonded to silicon, nitrogen, and/or hydrogen, i.e., completely embedded in the SiCN network. The second category, labelled “dimers”, corresponds to C–C pairs. The “chains” category describes linear chains of at least three carbon atoms, and the “branched chains” category contains chains with at least one ramification. The final category, called “sheets”, designates carbon structures containing at least one 3- to 7-atom carbon ring.

**Figure 5:**
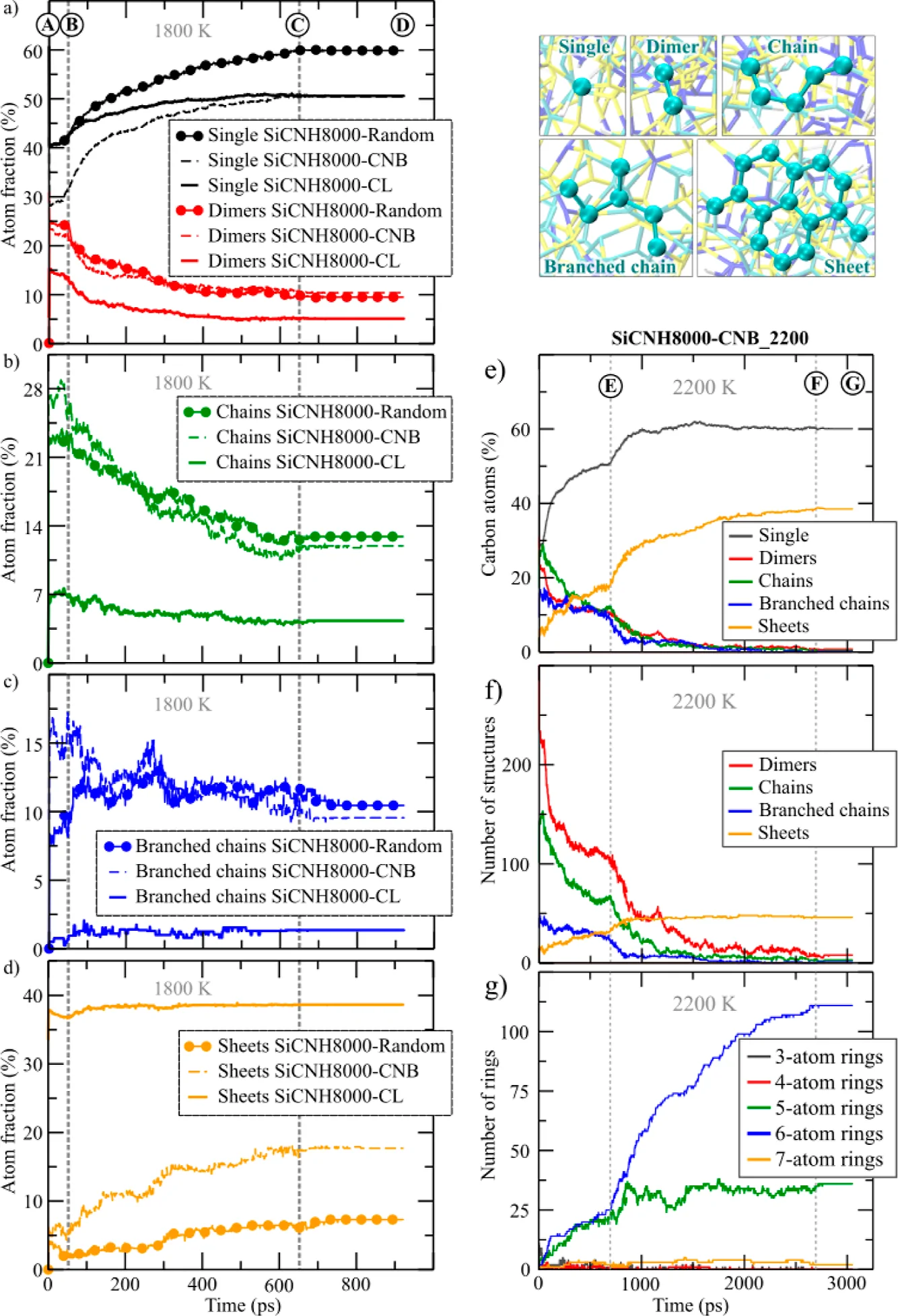
Evolution of the proportion of carbon atoms in different environments (single, dimers, linear chains, branched chains, and sheets) during the thermal cycle for the SiCNH8000-Random, SiCNH8000-CNB, and SiCNH8000-CL (a–d, left panels) and SiCNH8000-CNB_2200 (f). Evolution of (g) the number of specific carbon structures and of (h) the number of carbon rings with different sizes during the thermal cycle applied to SiCNH8000-CNB_2200. The vertical dashed lines delimit the highest temperature plateau for each thermal cycle. Circled A–G capital letters indicate the timing configurations shown in Figure 2a. The different types of carbon structures are illustrated (e) with examples extracted from the models.

Figures 5a–d and 5f show that the number of single C atoms increases significantly in all four models over time and becomes in all cases the main category at the end of the dynamics, with a fraction of approximately 50% to 60% in line with the formation of the SiCN network. Conversely, the fractions of atoms belonging to dimers and linear chains decrease steadily, with the most significant decrease occurring during the high-temperature plateaus, as observed visually in Figure S8 in the Supporting Information. The evolution of the fractions of atoms belonging to branched chains, however, depends on the specific model. In the SiCNH8000-Random and SiCNH8000-CL models, the fractions grow steadily during the first 105 ps of the annealing cycle before stabilizing around 11 and 3%, respectively, whereas they fluctuate with a global decrease down to 9% during the cooling phase in the SiCNH8000-CNB model. Finally, the fraction of carbon atoms belonging to sheets, already high by construction at the initial stage in model SiCNH8000-CL, is almost constant around 39%, seemingly due to the lack of available carbon in the direct surroundings of the preconstructed sheets.^40^ In contrast, this fraction increases steadily during the high-temperature plateau, from 2 to 6% in model SiCNH8000-Random and from 6 to around 17% in model SiCNH8000-CNB. As for SiCNH8000-CNB_2200 (Figure 5f,g), we observe a faster disappearance of dimers and linear/branched chains in favor of isolated atoms and sheets at the beginning of the 2200 K plateau. After *t* = 300 ps at this temperature, the growth of sheets and the isolated carbon atoms in the SiCN network exhibit a tendency to slow down.

We now focus on the evolution of carbon ring population in the models as shown in Figures S9 and 5h. For SiCNH8000-Random and SiCNH8000-CNB models, we observe an increase of the number of 5- and 6-member rings over time, with the latter showing a faster growth rate. In addition, the 3- and 4-member rings almost disappear during the very first stage of annealing (≈ 50 ps). In these systems, 7-member rings remain marginal during the annealing. The evolution in model SiCNH8000-CL differs significantly, due to the presence of 6-member carbon sheets in the initial configuration. During the dynamics, only 4 additional six-atom rings are formed at the periphery of pre-existing sheets. It is also notable that during the dynamics, 9 five-atom rings form after 100 ps. These rings are found at the border of the already existent sheets, inducing a slight growth of these domains.

Coming to the best SiCNH8000-CNB_2200 model, except at the beginning of the *T* = 2200 K plateau, we observe that the number of 5-member rings is constant (Figure 5h), whereas the number of 6-member rings undergoes a very rapid increase leading to the formation of carbon sheets dominated by 6-member rings. We note that, at the same time, dimers, chains, and branched chains disappear, which suggests that they contribute to the growth of the extended carbon sheets. Interestingly, as the number of sheets remains constant, this trend implies a growth of the layers instead of the increase of their number (Figure 5g,h). To complement this connectivity-based analysis, we examined the geometry of the evolving carbon phase through the C–C–C bond-angle distribution (BAD) (see Figure S10 and Section S12 in the Supporting Information for details). The distributions mainly extend between approximately 105° and 135°. During the *T* = 2200 K plateau, the relative intensity around 120° progressively increases, indicating an increasing prevalence of trigonal-planar, sp^2^-like carbon environments as the carbon sheets grow. During the subsequent cooling stage, this feature becomes sharper, consistent with an additional reduction of thermal broadening. At the end of the thermal annealing cycle, sheets representative of the free-carbon phase gather about 38% of the carbon atoms (noticeably close to the amount of sp^2^ carbon atoms in the model according to Table S7), while only 2% persist inside dimers or chain-like structures. The remaining 60% of carbon atoms are involved in the formation of the SiCN network. The PDC global composition can thus be decomposed into 40% of free carbon and 60% of C within the SiCN matrix with a composition Si_32_C_15_N_24_ (without accounting for H). This matrix composition is close to Si_32_C_14_N_24_, consistent with a mixture of 70% SiC and 30% Si_3_N_4_ that can be expressed with the following equation:

1 $${Si}_{32}C_{25}N_{24}\approx 10C+20{\left(SiC\right)}_{0.7}{\left({Si}_{3}N_{4}\right)}_{0.3}$$

This resulting chemically consistent phase separation further supports the ability of extended machine-learning molecular dynamics at sufficiently high temperatures to properly model these complex SiCN systems.

#### 2.4.2 Nucleation and Growth Mechanisms

Figure 6a–f shows local atomic configurations in the SiCNH8000-Random model at different times during the *T* = 1800 K plateau, highlighting the formation of an aromatic C ring. Starting from single carbon atoms and carbon dimers initially dispersed in the amorphous ceramic network (Figure 6a), we observe the progressive formation of a linear chain of 6 atoms through the coalescence of the initial structures (Figure 6b–d). The formation of additional C–C bonds allowing the growth of a carbon chain and eventually a ring is enabled by the breaking of Si–C or C–N bonds, with Si atoms bonding to nearby C or N atoms and N atoms to nearby Si atoms. The 6-atom linear chain (Figure 6d) closes to form a 6-member ring (Figure 6e), which later captures an additional carbon atom at its periphery (Figure 6f), potentially initiating further sheet growth through the construction of an additional adjacent ring. Similar trends were reported by Imoto et al. using metadynamics simulations, where carbon atoms first form polymer-like chains, followed by rings, and eventually hexagonal, graphene-like structures.^40^

**Figure 6:**
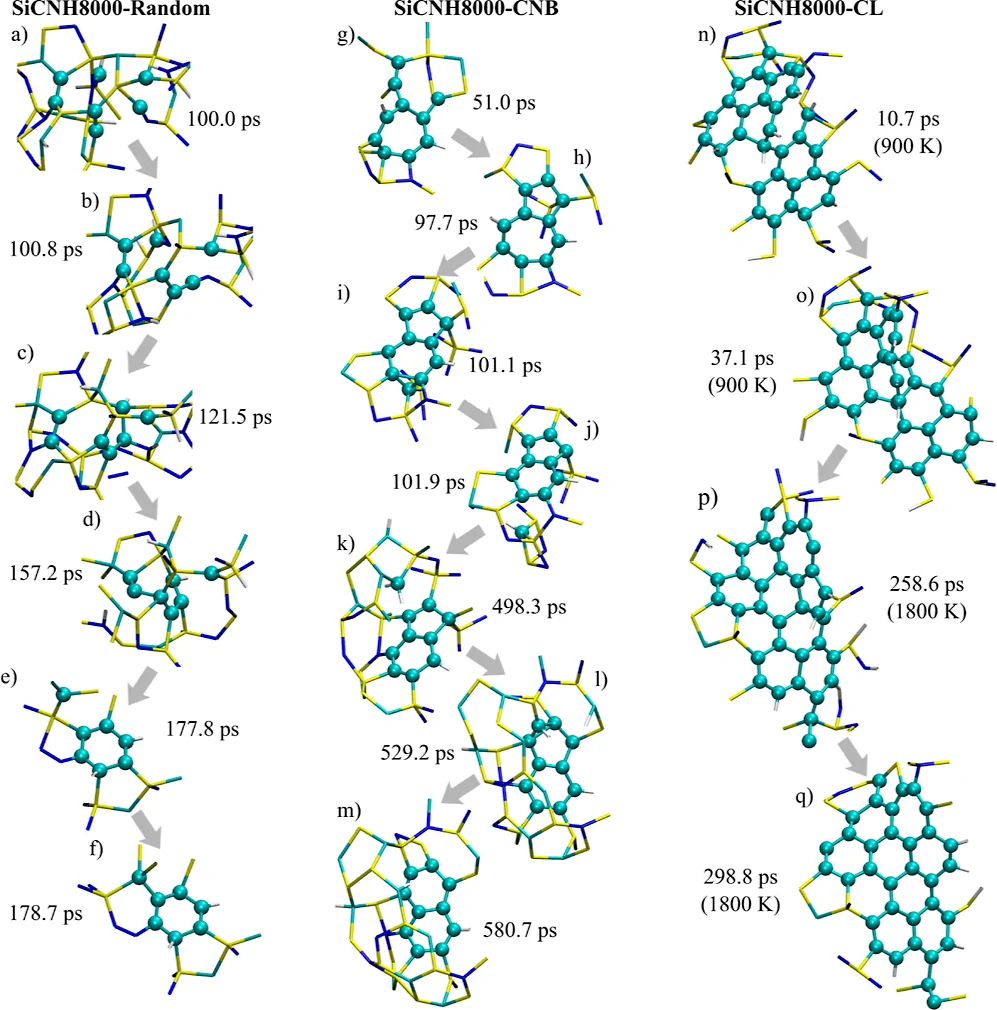
(a–f) Atomic configurations illustrating the mechanism of aromatic ring formation from isolated carbon atoms in the SiCNH8000-Random model. The spheres represent carbon atoms that are part of the final ring, highlighting the progressive assembly of dispersed carbon atoms into organized aromatic structures. (g–m) Atomic configurations illustrating the mechanisms of 6-member aromatic ring formation from 5- and 7-member rings in the SiCNH8000-CNB model. The spheres represent carbon atoms that are part of the final ring, demonstrating the transformation pathways between different ring structures. (n–q) Atomic configurations illustrating the coalescence of two carbon sheets in the SiCNH8000-CL model. The spheres represent carbon atoms that are part of the final sheet, demonstrating the complex process of sheet merging and structural reorganization. Yellow, blue, and white lines represent bonds made by the silicon, nitrogen, and hydrogen atoms, respectively.

As highlighted in Figures S9 and 5, rings containing 3, 4, 5, or 7 carbon atoms occasionally appear during the dynamics, among which only 5-member rings may be preserved throughout the annealing cycle. These imperfect cycles play an important transient role in the formation of six-atom aromatic cycles and the growth of carbon sheets, as illustrated in Figure 6g–m, showing atomic configurations extracted from the thermal cycle applied to the SiCNH8000-CNB model. Configuration 6g, extracted at the beginning of the *T* = 1800 K plateau (t = 51 ps), contains a 7-atom carbon ring with two lateral branches made of 1 and 2 C atoms, which eventually bind to form an adjacent 5-atom ring (Figure 6h). Configurations 6i,j show one possible mechanism by which an aromatic 6-member ring is formed from a 7-atom ring. One of the C atoms is ejected from the initial ring via the formation of an evanescent 3-member ring, wherein the (later) ejected C atom first forms a C–C_2_Si_2_ tetrahedron. It then quickly leaves the vicinity of the carbon sheet by bonding to a third Si atom and one hydrogen atom to integrate the SiCN network, like the majority of carbon atoms in the SiCNH8000-CNB model (see Table 4).

The opposite mechanism is observed around the 5-atom ring later during the thermal cycle. Specifically, a C atom sitting in a C–CSiH_2_ tetrahedron, eventually positioned above two atoms of the 5-atom ring (Figure 6k), forms a 3-C ring (Figure 6l) similar to the one observed in Figure 6i, which then intercalates into the carbon ring to convert it into a stable aromatic 6-member cycle (Figure 6m).

While carbon sheet growth mainly occurs through the capture of single atoms or dimers, the coalescence of nearby sheets has also been observed in the SiCNH8000-CL model, as illustrated in Figure 6n–q. Configuration 6n, obtained after 10.7 ps (at *T* = 900 K), shows two carbon sheets made of 4 and 3 rings and connected by a shared lateral C–C bond. A 1-C atom branch located at the periphery of the 3-ring sheet binds, in configuration 6o, with the 4-ring sheet to form a 6-atom cycle, wherein three atoms are shared with a 7-atom ring created by the approach of the two initial sheets. This resulting extended carbon structure contains C atoms located above its main plane, which then progressively move to its periphery to obtain a nearly planar structure (Figure 6p). An aromatic cycle is finally formed, leading to a single large sheet composed of 7 6-member and 1 7-member rings (Figure 6q).

Taken together, Figure 6a–q highlights the mechanisms of nucleation and growth of free-carbon domains in Si_32_C_25_N_24_H_19_ models, wherein ring formation appears to be a strongly defect-mediated process, with short-lived 3-, 4-, 5-, and 7-member rings playing key roles. This growth mechanism is in line with recent metadynamics simulation of carbon layer formation on the SiC(0001) surface, where it was shown that under-coordinated C reorganizes in lines, rings, and eventually aromatic seeds to ultimately form graphene.^40^

The extension of the dynamics and the temperature rise applied to the SiCNH8000-CNB_2200 model allowed us to continue the growth and the structuring of free-carbon domains initiated in the SiCNH8000-CNB model. During this high-temperature dynamics, nearly all dimers and carbon chains disappear, leaving carbon atoms in a phase-separated configuration: isolated in the SiCN network or belonging to graphitic sheets.

The nucleation and growth mechanisms described above should be regarded as a prediction of the simulations, since direct experimental observation of the individual bond-breaking, bond-forming, and ring-reorganization events involved in the phase separation of polymer-derived SiCNH ceramics is hardly accessible through experimental characterization that usually probes the structural evolution at a more global level or on the final ceramic. In particular, the progressive segregation of carbon from the SiCN ceramic network and its organization into increasingly extended sp^2^-rich domains are well-established features of the thermal evolution of polymer-derived SiCN ceramics.^12,14^

Importantly, similar microscopic processes are observed across several trajectories and in models that start from different initial configurations. These processes include the nucleation and growth of carbon rings, transformations involving defective non-six-membered rings, incorporation of nearby carbon atoms or small carbon fragments, and coalescence of neighboring carbon domains. Their repeated observation in independent trajectories supports the robustness of the proposed atomistic picture. Direct experimental verification of the individual elementary steps nevertheless remains an important goal for future studies. Local environment-sensitive techniques, such as solid-state NMR, could provide further validation by comparing the experimental and computed NMR spectra.

## 3 Conclusions

In this work, we present a generic methodology for constructing a highly accurate machine learning interatomic potential for polymer-derived hydrogen-containing silicon carbonitride (SiCNH) ceramics. To capture the full complexity of amorphous Si–C–N–H systems, our approach combines a meticulously constructed training database containing FPMD amorphous models and diversified with crystal structure prediction models, together with advanced machine learning architectures. The obtained MACE potential shows an accuracy of 12.4 meV/atom for energies and 149 meV/Å for forces, with strong transferability across diverse structural environments and compositions.

Large-scale molecular dynamics simulations of 8000-atom systems of composition Si_32_C_25_N_24_H_19_ with various initial configurations and thermal cycles led to four PDC models. Compared to PDF experiments, the best model is unambiguously the one that spontaneously evolves toward a clear phase separation, characterized by dispersed carbon sheets embedded in an amorphous SiCN ceramic network, starting from an appropriately tuned initialization and given a long enough simulation time at a temperature deliberately higher than experimental pyrolysis temperature. For the considered PDC composition, we find that about 40% of the carbon atoms belong to the sheets, while the remaining 60% of carbon atoms participate in the formation of the SiCN network. The latter is mainly built of 4-fold silicon atoms bonded to both nitrogen and carbon atoms, 4-fold carbon atoms mainly bonded to silicon atoms, and finally 3-fold nitrogen atoms. The occurrence of this biphasic system requires long thermal annealing at high temperatures (*T* = 2200 K). It brings the calculated atomic pair distribution function into excellent agreement with experimental data, particularly in the medium-range order, suggesting a similar carbon distribution in the real sample.

The structuring of the free-carbon phase goes along with the disappearance of carbon dimers and small carbon chains, as well as the growth of carbon sheets containing mainly aromatic rings. The nucleation and growth of these carbon sheets are mediated by defective carbon rings, which serve as intermediate structures that facilitate the formation of stable 6-member aromatic cycles through the capture of additional carbon atoms or the ejection of excess ones to optimize local bonding configurations.

The present simulations also show that temperature strongly affects the rate of carbon-phase organization with the 2200 K treatment promoting the growth of larger carbon sheets. However, as the thermal cycles used here were designed to reveal the phase-separation mechanisms within accessible simulation times, a systematic study of temperature, heating/cooling rates, and plateau durations would therefore be of interest to establish a full processing-structure relationship.

The convergence of our models toward experimentally observed structures, regardless of their initial configurations, underscores the thermodynamic robustness of our potential and its ability to capture the intrinsic structural features of these complex ceramics and the mechanisms of free-carbon-phase growth. Beyond the specific composition studied in this work, this methodology can be applied to carbon-rich PDC systems to study the formation of extended turbostratic carbon structures.

## 4 Methods

### 4.1 Database Construction

The database used for training the MLIP consists of seven distinct classes of configurations, carefully selected to represent the structural diversity of Si–C–N–H systems (Table 5). It contains over 9000 configurations spanning a wide range of compositions, densities, structural motifs, and networking. The relatively large size of the database reflects the broad structural and compositional diversity required to describe the reactive Si–C–N–H energy landscape rather than a need to stabilize individual MD trajectories.

**Table 5:** Composition of the Training Database for the Si–C–N–H MLIP

class	chemical system	atom count	number of configurations
SiCNH-periodic	Si–C–N–H	200 and 400	4033
SiCNH-periodic-4000K	Si–C–N–H	400	1497
SiCNH-surface	Si–C–N–H	200 and 400	2000
amorphous-carbon-200	C	216	247
amorphous-carbon-250	C	216	249
amorphous-silicon	Si	512	250
crystal-structure-prediction	Si–C–N–H	46 to 100	1165
total			9441

To ensure the consistency of the database, the energies and forces of all the collected configurations were recalculated by resorting to the CP2K code^41^ using the same density functional theory (DFT) parameters with the PBE functional,^42^ GTH pseudopotentials,^43^ double-ζ polarized basis sets,^44^ a plane-wave energy cutoff of 1000 Ry, and semi-empirical Grimme’s D3 dispersion corrections.^45^ For smaller systems (46–100 atoms) obtained from crystal structure prediction (using a different DFT code, as will be detailed below), Γ-point calculations are not sufficient. These systems were therefore subjected to a single-point energy calculation in CP2K, using the same parameters as the FPMD described above, with the addition of *k*-point meshes constructed automatically to ensure a maximum spacing of 0.3 Å^–1^ between *k*-points in the reciprocal space.

#### 4.1.1 Configurations Derived from First-Principles Molecular Dynamics

The SiCNH-periodic class contains configurations extracted from FPMD trajectories of periodic models with the composition Si_32_C_25_N_24_H_19_. This class includes configurations from seven models presented in our previous work^27^ in addition to three models produced with the purpose of increasing amorphous configuration diversity. Details of the three additional models are provided in the Supporting Information paragraph S1. 125 configurations were sampled from each temperature plateau of the specific thermal cycles applied to every model (the temperature ranging from 300 to 1800 K). It results in a wide database of more than 7000 configurations, from which approximately 4000 are selected to feed the training, validation, and test sets employed to develop the MLIP.

The SiCNH-periodic-4000K class contains high-temperature configurations (up to *T* = 4000 K) generated from model SiCNH400-A of ref 27 to sample high-energy states. This class is particularly important for ensuring that the MLIP can accurately describe configurations far from equilibrium, which may be encountered during phase separation and thermal processing. The high-temperature configurations were generated using the thermal cycle presented in Figure S1 in the Supporting Information, resulting in 1497 configurations.

The SiCNH-surface class contains configurations from four surface models, with and without exposed carbon layers/chains, produced using the procedure described in the Supporting Information paragraph S3. Similar to the periodic models, 125 configurations were sampled from each temperature plateau (*T* = 1300, 900, 600, and 300 K), resulting in 2000 configurations. The inclusion of these surface configurations aims to capture the structural motifs that may appear at interfaces or in the vicinity of free-carbon domains.

#### 4.1.2 Amorphous Carbon and Silicon Configurations

The Amorphous-Carbon-200 and Amorphous-Carbon-250 classes incorporate amorphous carbon structures at densities of 2.00 and 2.50 g/cm^3^, respectively. Initial configurations were obtained from the work of Deringer and Csányi,^46^ where melt-quenched models were produced at different densities, resulting in models with carbon sp, sp^2^, and sp^3^ hybridizations. Each initial amorphous carbon configuration was subjected to a short thermal cycle of about 20 ps at *T* = 300 K, from which 247 and 249 configurations were extracted from Amorphous-Carbon-200 and Amorphous-Carbon-250 models, respectively.

Similarly, for the amorphous-silicon class, we collect the initial configuration from the work of Deringer et al.^47^ Specifically, we consider the model containing 512 silicon atoms obtained from a melt-quenching simulation with a quenching rate of 10^11^ K/s. The initial configuration was subjected to a short thermal cycle of 20 ps at *T* = 300 K, from which 250 configurations were included into our database.

The inclusion of these configurations is of paramount importance for describing the carbon- or silicon-rich environments that may be encountered in certain regions of the SiCN ceramic network and particularly the free-carbon domains that may nucleate and grow during a phase separation.

#### 4.1.3 Crystal Structure Predictions

The Crystal-Structure-Prediction class contains 1165 configurations generated using the USPEX program^48–50^ for various Si–C–N–H compositions and densities. USPEX is a crystal structure prediction (CSP) program employing an evolutionary algorithm to generate, generation after generation, a diverse set of candidate structures for a given composition. While the purpose of this approach is generally to efficiently explore the potential energy surface and determine the most stable crystalline structure, here we exploit, instead, the structural diversity resulting from this exploration. Each structure generated with the evolutionary algorithm is relaxed with DFT, using the VASP code,^51,52^ within 3 consecutive steps of increasing numerical accuracy, followed by a high-accuracy single-point energy calculation (see the Supporting Information paragraph S4 for details).

The CSP configurations were generated for seven distinct compositions representing variations around the target material composition, with some compositions independently explored at optimized and (fixed) low density (see Table 6). The low-density configurations were generated by fixing the volume to 1.25 times the average volume of the 10 best structures from variable-density calculations conducted for the same composition. For each composition and density, 1000 to 2000 structures were generated, and 100 to 200 were selected to build the database (see Supporting Information paragraph S4).

**Table 6:** Configurations Generated by Crystal Structure Prediction (Crystal-Structure-Prediction Class)

name	composition	number of configurations	
		optimized density	low density
Ref-compo	Si_16_C_12_N_12_H_10_	110	0
Ref-compo-low-density	Si_32_C_24_N_24_H_20_	0	144
C-rich	Si_12_C_20_N_10_H_8_	120	0
C-poor	Si_16_C_8_N_12_H_10_	115	0
N-rich	Si_12_C_10_N_20_H_8_	114	0
N-poor	Si_18_C_14_N_6_H_12_	104	0
H-rich	Si_12_C_9_N_9_H_20_	139	131
C–H-rich	Si_8_C_16_N_8_H_18_	104	84

The inclusion of these crystalline configurations is crucial for ensuring that the MLIP can accurately describe structures that may be far from the typical amorphous configurations encountered in the FPMD simulations. This diversity is particularly visible in the multidimensional scaling^53,54^ 2D projection of the training database using Valle–Oganov fingerprints^55^ as given in Figure 7. It is very important for capturing the complex energy landscape of Si–C–N–H systems and ensuring the transferability of the potential across different structural motifs.

**Figure 7:**
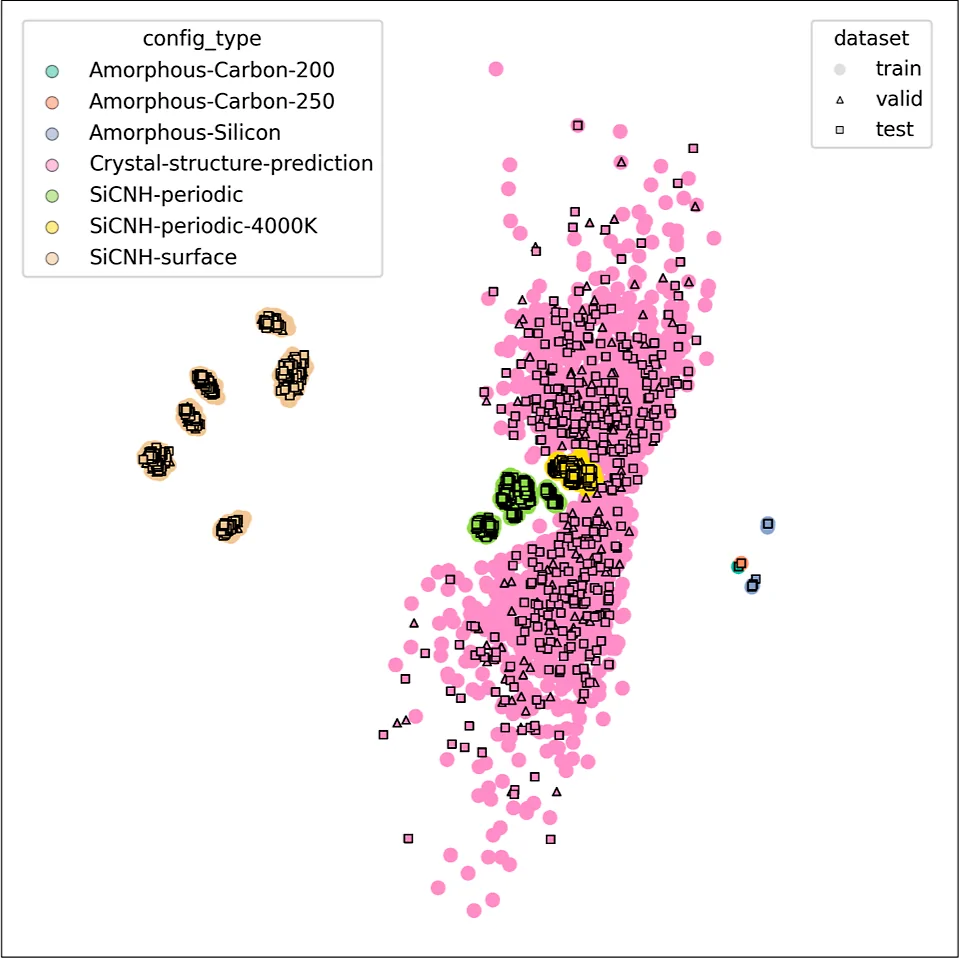
Multidimensional scaling 2D projection of the training database using Valle–Oganov fingerprints,^55^ demonstrating the structural diversity captured by the database. Each point represents a configuration, with colors indicating different classes of structures.

### 4.2 MLIP Training

The interatomic potential was developed using the MACE framework^31,32^ (version 0.3.9), which implements a message-passing neural network architecture that has shown good performance for a variety of materials systems.^56–58^ The architecture consists of 2 message-passing layers with 128 scalar (*L* = 0) and 128 vector (*L* = 1) components, providing a balance between computational efficiency and descriptive power. The correlation order ν was set to 3, allowing the potential to capture 4-body interactions (one central atom and three neighbors). Such level of correlation aims at accurately describing the complex bonding environments in Si–C–N–H systems, where the coordination of silicon and carbon atoms can vary significantly depending on the local chemical environment. The atomic cluster expansion descriptor is used to represent atomic environments and uses spherical harmonics up to degree *l*_max_ = 3 and 8 radial basis functions derived from 5 Bessel functions. The cutoff radius was set to 5.5 Å, which corresponds to an effective receptive field of 11 Å, thereby providing a good balance between capturing long-range interactions and maintaining computational efficiency. The model training was performed using an Adam optimizer^59^ with a batch size of 10 configurations.

Given the complexity of the explored energy landscape, the training was conducted gradually in five successive steps, wherein the training database was progressively expanded, as shown in Table 7. At each step, training was resumed from the checkpoint obtained at the end of the previous step by using the enlarged database. The epoch numbering shown in Figure 8 is therefore cumulative over the five training steps. This gradual procedure allows the potential to first learn the basic features of the energy landscape before being exposed to more specific and complex configurations.

**Table 7:** Training Steps for the Si–C–N–H MLIP

class	DB-01	DB-02	DB-03	DB-04	DB-05
SiCNH-periodic	X	X	X	X	X
SiCNH-surface					X
SiCNH-periodic-4000K				X	X
amorphous-carbon-200			X	X	X
amorphous-carbon-250	X	X	X	X	X
amorphous-silicon		X	X	X	X
crystal-structure-prediction	X	X	X	X	X

**Figure 8:**
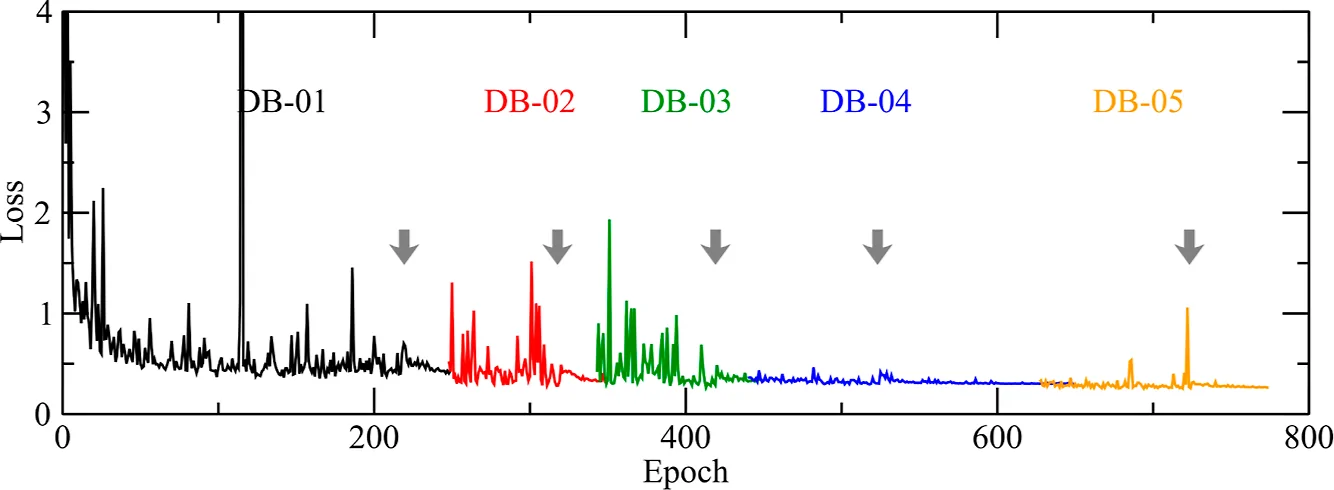
Evolution of the loss on the validation set during the training process. The five successive training steps DB-01 to DB-05 use progressively enlarged databases, with the epoch number shown cumulatively. Within each training step, the initial stage focuses on force optimization and is followed by the SWA/energy-refinement stage. Arrows indicate the transition to the SWA stage.

Each training step consisted of two stages: an initial stage focusing on force optimization, followed by an SWA stage focusing on energy refinement. In practice, this is governed by the modification of the learning rate and the weights of the loss function L given in eq 2.

2 $$L=\frac{\lambda_{E}}{B}\sum_{b=1}^{B}{\left(\frac{E_{b}-{Ê}_{b}}{N_{b}}\right)}^{2}+\frac{\lambda_{F}}{3B}\sum_{b=1}^{B}\sum_{i_{b},\alpha =1}^{N_{b},3}{\left(-\frac{\partial E_{b}}{\partial r_{i_{b},\alpha }}-{\mathrm{F̂}}_{i_{b},\alpha }\right)}^{2}$$

Here, L corresponds to a weighted sum of the mean square errors of the total energy and forces components over the atomic configurations *b* containing *N*_*b*_ atoms in a batch of size *B*. *E*_*b*_ depicts the energy predicted by the model, while ${Ê}_{b}$ and ${\mathrm{F̂}}_{i_{b},\alpha }$ correspond to the DFT configuration energy and force components of the atom *i* of configuration *b* along the α direction, respectively. During the force optimization stage, the learning rate was set to *lr* = 0.01, with loss weights of λ_*F*_ = 100.0 for forces and λ_*E*_ = 1.0 for energies, and during the energy refinement stage, the learning rate was reduced to *lr* = 0.001, with loss weights of λ_*F*_ = 100.0 for forces and λ_*E*_ = 1000.0 for energies. This two-stage approach allows the potential to first adjust the model hyperparameters with less tight constraints before refining them to describe the subtle energy differences between configurations.

Furthermore, each database introduced at a given training step is first split into 80% training–validation and 20% test sets. This ensures that 20% of both the whole dataset and of each individual database (SiCNH-periodic, SiCNH-surface, CSP, etc.) remain totally unseen by the training process. Subsequently, the training–validation set is split into 90% training and 10% validation sets. The training set is used for training the model, while the validation set is used to monitor the validation loss during the training and prevent overfitting. The final model performance was evaluated on the totally unseen test set.

The training process was conducted over 775 epochs. Figure 8 shows the evolution of the loss function on the validation set during training, with arrows indicating the transition from the force-optimization to the energy-refinement stage for each training step. Figure S4 in the Supporting Information displays the evolution of the associated root mean square errors (RMSEs) for energies and forces. In both training stages, force errors decrease at first, followed by a more gradual improvement in the energy prediction accuracy.

### 4.3 Structural Model Generation

Molecular dynamics simulations were performed using the LAMMPS software.^60^ LAMMPS was configured with the corresponding mace pair style and calculations were performed on 1 GPU, enabling simulation of thermal cycles lasting for a few nanoseconds on systems of 8000 atoms. To fully exploit modern GPU architectures, we compiled LAMMPS with Kokkos + CUDA^61^ and Message Parallel Interface (MPI), explicitly targeting the NVIDIA H100 architecture. All simulations were conducted in the *NVT* ensemble using periodic boundary conditions, a time step of 0.5 fs and thermal control through a Nosé–Hoover thermostat,^62–64^ ensuring an optimal balance between computational efficiency and accurate integration of the equations of motion.

Four models of composition Si_32_C_25_N_24_H_19_ were generated based on three initial configurations produced at the experimental density of 2.17 g/cm^3^, corresponding to a cubic cell of side length of around 4.5 nm, using different approaches to investigate the influence of the initial atomic arrangement on the final structure after thermal annealing. This composition has been extensively studied in our previous FPMD work, using models up to 400 atoms,^27^ which provided the primary brick of our database.

The initial configuration of the **SiCNH8000-Random** model corresponds to a random distribution of atoms generated using the Packmol software,^65,66^ without imposing a priori constraints on the network connectivity. This deliberately unconstrained configuration is used as a reference to assess the influence of the initial atomic organization on the subsequent structural evolution and allows us to assess the ability of the MLIP to describe highly random structures.

The SiCNH8000-CNB configuration was generated using the ceramicNetworkBuilder program with parameters that were explored and adjusted in our previous work.^27^ This approach leads to an amorphous network based on specified pair bonding and coordination probabilities, providing a more realistic starting point than the random configuration. We here used parameters that favor the formation of small carbon motifs (chains and clusters) in the initial model. The target bonding and coordination number probabilities employed to generate the initial configuration of the SiCNH8000-CNB model are given in Tables S2 and S3 in the Supporting Information

For the **SiCNH8000-CL** configuration, prior to generating the amorphous network, 46 carbon sheets (27 with 3 rings and 19 with 4 rings) were inserted into the simulation cell, representing 32.5% of the carbon atoms in the system. Next, the ceramicNetworkBuilder program was used to complete the amorphous network (see parameters in Tables S2 and S3 in the Supporting Information). Carbon atoms outside of the pre-formed carbon layers were forced to bond only to silicon in order to diversify the population of SiC_*x*_N_4–*x*_ tetrahedra in the amorphous network of the ceramic, by creating more Si–C_2_N_2_ or Si–C_3_N units as evidenced by NMR analysis.^39^ This approach provides a starting point with pre-formed carbon domains, allowing us to study their evolution during thermal cycling.

Figure S6 in Supporting Information shows the initial configurations of the three models. The distribution of carbon atoms varies significantly between the models, from isolated atoms and small clusters in the SiCNH8000-Random model to extended pre-formed rings and clusters in the SiCNH8000-CL model.

The three starting configurations were subjected to the same thermal cycle using MLIP molecular dynamics (Figure 2a). It consists of subsequent temperature plateaus separated by 2 ps transitions to ensure that there are smooth temperature changes. The cycle started with a first stabilization phase of 3 ps at *T* = 300 K, followed by a heating phase consisting of 2 ps at *T* = 600 K, 18 ps at *T* = 900 K, 8 ps at *T* = 1100 K, 10 ps at *T* = 1500 K, a long plateau of 600 ps at the maximum temperature of *T* = 1800 K, a cooling phase consisting of 40 ps at *T* = 1500 K, 40 ps at *T* = 1200 K, 40 ps at *T* = 900 K, and 40 ps at *T* = 600 K, and finally 100 ps at *T* = 300 K. This thermal cycle is designed to study the structural evolution of the SiCNH material during annealing phases within reasonable simulation times.

To favor possible growth of the carbon domains, we extended the thermal cycle for the SiCNH8000-CNB model, starting from the end of the *T* = 1800 K plateau to reach a temperature of *T* = 2200 K (via an intermediate plateau at *T* = 2000 K) and maintained this elevated temperature for 2 ns, after which the system was brought down to room temperature. The fourth model obtained at the end of this extended thermal cycle is henceforth termed SiCNH8000-CNB_2200.

## Supplementary Material



## Data Availability

The reference database and trained MACE potential developed in this work, released as IRCER-PDC-DB v1.0 and IRCER-SiCNH-MACE v1.0, respectively, are publicly available on GitHub at https://github.com/ASM2C-group/IRCER-PDC-MLIP.

## Notes

Additional periodic models, high-temperature and surface-model generation protocols, crystal structure prediction and model training, dynamical validation of the MLIP against first-principles molecular dynamics, initial parameters of the 8000-atom models, PDF scaling and comparison, atomic local-environment analyses, and additional analyses of carbon-domain evolution, carbon-ring populations, and C–C–C bond-angle distributions (PDF)

## Notes

**Preprint** A preprint version of this work is available as Mortier, F.; Cadars, S.; Masson, O.; Boero, M.; Ori, G.; Wang, Y.; Bernard, S.; Bouzid, A. Modeling phase separation in polymer-derived silicon carbonitride ceramics through extended machine learning molecular dynamics. 2026, 2605.20358. arXiv. https://arxiv.org/abs/2605.20358 (accessed September 23, 2026).
