# Supplementary material for: Modeling Phase Separation in Polymer-Derived Silicon Carbonitride Ceramics through Extended Machine Learning Molecular Dynamics
Source: ACS Nano. 2026 Oct 6;20(41):27874–89. doi: 10.1021/acsnano.6c11958 (PMC13689924; doi:10.1021/acsnano.6c11958)
Supplement: Supplementary file 1 [file nn6c11958_si_001.pdf]

Modeling phase separation in polymer-derived silicon carbonitride ceramics through extended machine learning molecular dynamics

Fabien Mortier,[†] Sylvian Cadars,[†] Olivier Masson,[†] Mauro Boero,[‡,¶] Guido Ori,[¶,§] Yun Wang,[‖] Samuel Bernard,[†] and Assil Bouzid[*,†]

†*Institut de Recherche sur les Céramiques, UMR 7315 CNRS-Université de Limoges, Centre Européen de la Céramique, 12 rue Atlantis 87068 Limoges, France*

‡*University of Strasbourg, CNRS, ICube Laboratory UMR 7357, F-67412 Illkirch, France*

¶*ADYNMAT CNRS consortium, F-67034, Strasbourg, France.*

§*Université de Strasbourg, CNRS, Institut de Physique et Chimie de des Matériaux de Strasbourg, UMR 7504, F-67034 Strasbourg, France*

‖*School of Environment and Science, Griffith University, Gold Coast 4222, Australia*

E-mail: assil.bouzid@cnrs.fr

S1. Additional periodic models

Three additional periodic models, of composition $Si_{32}C_{25}N_{24}H_{19}$, containing 400 atoms are constructed to enrich the database of amorphous SiCNH systems.

The first model is produced using Born-Oppenheimer Molecular Dynamics (BOMD) as implemented in the CP2K suite of programs[1]. The calculations were conducted in the NVT ensemble in which the temperature is monitored by a Nosé-Hoover thermostat chain[2–5]. A timestep of 1 fs was used to integrate the equations of motion. The initial atomic configuration is generated with the ceramicNetworkBuilder program, employing bonding and coordination probabilities identical to those used for the SiCNH200 and SiCNH400-C models in Ref.[6]. Specifically, Si atoms have a 0% probability of bonding with Si, a 10% probability of bonding with C, and a 90% probability of bonding with N. The coordination probabilities are set such that C atoms have a 75% probability of 3-coordination, while Si atoms have a 100% probability of 4-coordination and N atoms have a 100% probability of 3-coordination. The thermal cycle applied during BOMD simulations includes the following stages: 4 ps at 600 K, 7 ps at 1300 K, 7 ps at 1800 K, 10 ps at 1300 K, 5 ps at 900 K, 5 ps at 600 K, and 15 ps at 300 K. This cycle allows the system to explore a wide temperature range, ensuring thorough relaxation.

The second model follows the same procedure as the SiCNH400-CL-3 model in Ref.[6]. Initially, three graphene-like sheets, each consisting of 4 carbon rings, are inserted into a periodic cell. The ceramicNetworkBuilder program code is then used to construct the amorphous SiCNH network around these sheets, achieving a final composition of $Si_{32}C_{25}N_{24}H_{19}$. The bonding probabilities for this model are set such that Si atoms have a 0% probability of bonding with Si, a 25% probability of bonding with C, and a 75% probability of bonding with N. C and N atoms have a 100% probability of 3-coordination, and Si atoms have a 100% probability of 4-coordination. After generating the initial configurations, a geometry optimization step is performed, followed by a thermal cycle using the second-generation Car-

Parrinello molecular dynamics (SGCPMD)[7,8]. The thermal cycle for this model includes: 4 ps at 300 K, 4 ps at 600 K, 20 ps at 900 K, 10 ps at 1100 K, 12 ps at 900 K, 12 ps at 600 K, and 30 ps at 300 K.

The third model, named SiCNH400-CL-4, is generated using a procedure similar to that of SiCNH400-CL-3 model in Ref.[6]. The only difference is that it incorporates two sheets of seven carbon rings dispersed within the amorphous SiCNH network. The final atomic configuration is achieved after applying the following second-generation Car-Parrinello molecular dynamics annealing thermal cycle: 4 ps at 300 K, 4 ps at 600 K, 20 ps at 900 K, 10 ps at 1100 K, 12 ps at 900 K, 12 ps at 600 K, and 30 ps at 300 K.

S2. Thermal cycle used for generating the high-temperature configurations

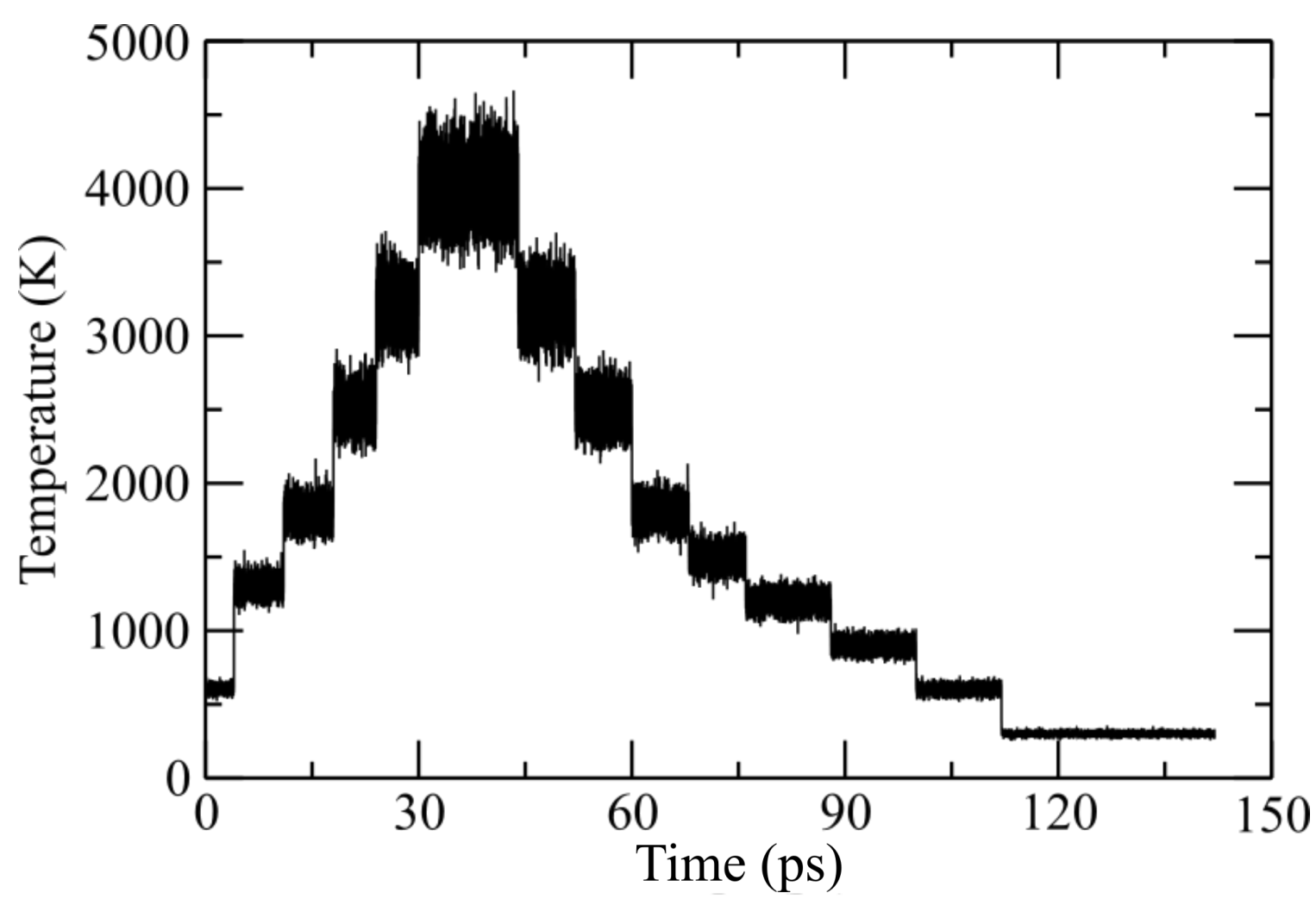

Figure S1: Thermal cycle used for generating the high-temperature configurations in the SiCNH-periodic-4000K class.

S3. Surface models generation protocol

Starting from periodic 400-atom models, the periodicity of the simulation cell along the z direction was broken by inserting a vacuum layer of approximately 20 Å. After a DFT-geometry optimization step, the surfaces are stabilized using a thermal cycle with the SGCPMD scheme.

MD simulations are conducted within an NVT ensemble with a timestep of 1 femtosecond for integrating the equations of motion. In SGCPMD, the conservation of temperature and total system energy relies on adjusting the Langevin friction coefficients, denoted as γ_L and γ_D. The coefficient γ_L is fixed at 0.1 fs^{-1}, while γ_D is adjusted between 0.018 and 0.020 fs^{-1}, depending on the temperature target, to ensure the conservation of the total energy.

The thermal cycle applied to relax the ceramic surfaces follows a gradual sequence: 5 ps at T = 300 K, 5 ps at T = 600 K, 5 ps at T = 900 K, followed by 10 ps at T = 1300 K, then descending in steps of 5 ps at T = 900 K and T = 600 K, and finally 20 ps at T = 300 K. This protocol allows for exploration across a wide temperature range, promoting optimal relaxation of the structures while avoiding abrupt transitions that could compromise their stability.

S4. Crystal structure prediction

Table S1 gives the main parameters used to perform multi-stage relaxation and energy calculation with the VASP code for every structure generated by the crystal structure prediction algorithm. The Perdew-Burke-Ernzerhof (PBE) exchange and correlation functional[9] was used at all stages, with van der Waals interactions described with Grimme's DFT-D3 method with zero-damping function[10]. The maximum spacing between k-points in the table uses the VASP definition, wherein the number of k-points in a given direction i of the reciprocal space is given by $N_i = \max(1, \lceil |\mathbf{b}_i| 2\pi / k_{spacing} \rceil)$, which involves a 2π factor with respect to

the definition used in USPEX. This gives dense k-point meshes able to precisely describe potentially non-insulating systems in the last stages. Similar parameters were used for fixed-low-density CSP runs, with all relaxation steps performed with free shape but fixed volume, with a cutoff energy sequence of 350, 400 and 450 eV, (and the same value of 520 eV for the final energy calculation).

Table S1: Summary of computational parameters for atomic relaxation and energy calculations.

Step	Type	Elect.-energy threshold (eV)	Ionic-energy threshold (eV)	Volume/ shape	Cutoff energy (eV)	K-point spacing ($Å^{-1}$)	Gaussian smearing (eV)
1	relaxation	10^{-3}	10^{-2}	fixed	300	0.63	0.08
2	relaxation	10^{-4}	10^{-3}	free	390	0.38	0.06
3	relaxation	10^{-5}	10^{-4}	free	520	0.19	0.04
4	energy	10^{-6}	N.A.	N.A.	520	0.12	0.04

Different selection procedures were combined to select 100 to 200 structures in each USPEX run. Some structures were selected randomly, while others were selected by first truncating the energy range of all structures in a run into fragments and then trying to pick in each energy segment a structure as distinct as possible (in the sense of Valle-Oganov distances[11]) from the one selected in the energy-segment below as illustrated in Figures S2 and S3.

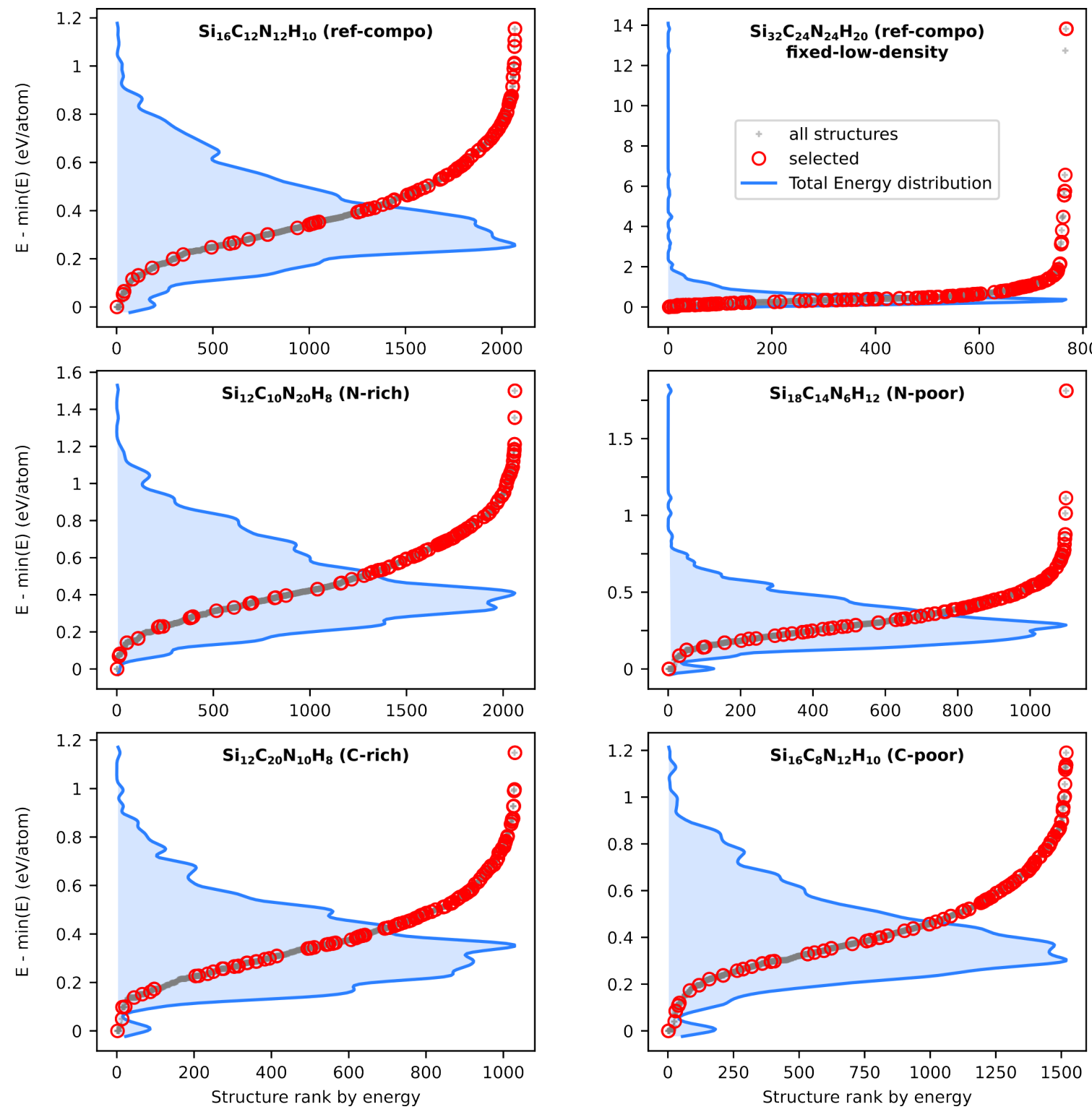

Figure S2: Relative energies of structures explored within different CSP runs (grey crosses), with the corresponding energy distribution shown in blue. Structures selected to feed the MLIP training database are shown as red circles.

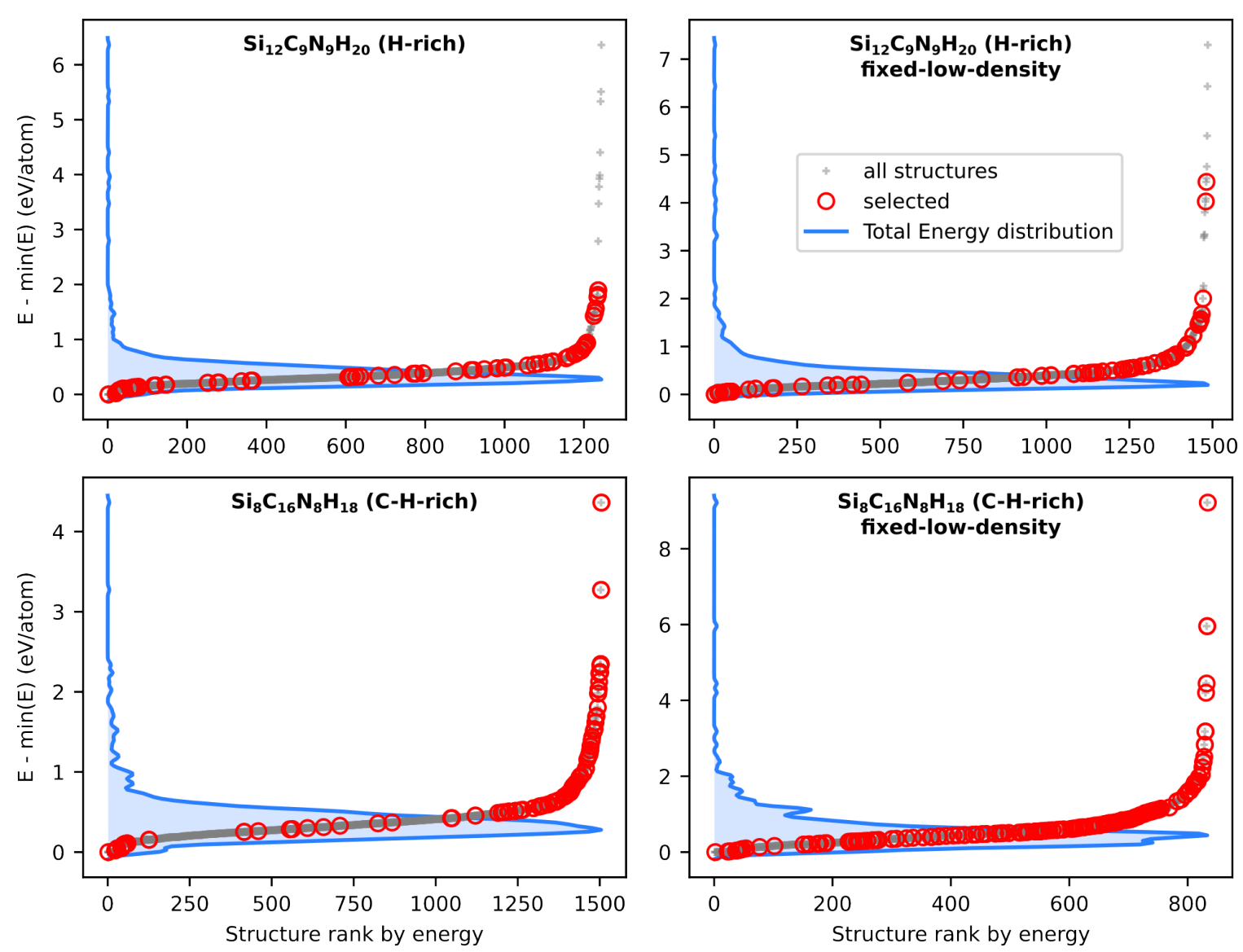

Figure S3: Relative energies of structures explored within other CSP runs (grey crosses), with the corresponding energy distribution shown in blue. Structures selected to feed the MLIP training database are shown as red circles.

S5. Model training

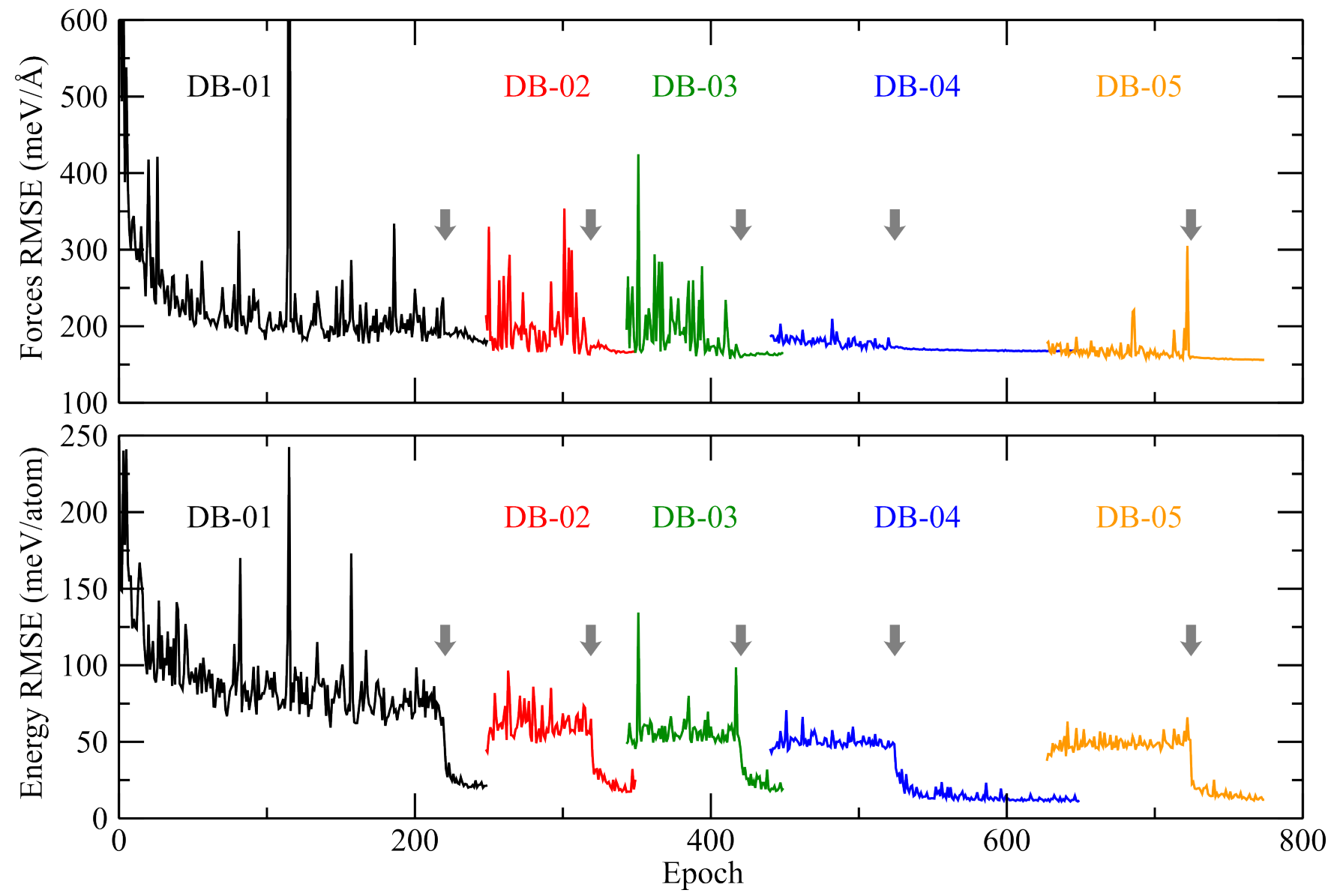

Figure S4: Evolution of root mean square errors (RMSE) for energies and forces on the validation set during the training process. Arrows indicate switches from the force-optimization to the energy-optimization stage.

S6. Dynamical validation of the MLIP against first-principles molecular dynamics

To complement the validation based on energies and forces, the dynamical accuracy of the MLIP was assessed through a direct comparison with first-principles molecular dynamics (FPMD). For this purpose, we considered the 200-atom SiCNH model previously investigated by FPMD in Ref.[6]. Starting from the same initial atomic configuration, an additional molecular dynamics trajectory of approximately 120 ps was generated using the MACE potential, following a thermal annealing protocol comparable to that employed in the FPMD simulation and that consisted of 5 ps at 300 K, followed by a 2 ps heating ramp from 300 to 600 K, 6 ps at 600 K, a 2 ps ramp from 600 to 900 K, 5 ps at 900 K, a 2 ps ramp from 900 to 1300 K, 10 ps at 1300 K, a 5 ps ramp from 1300 to 1800 K, and 10 ps at 1800 K.

The system was then cooled through a 5 ps ramp from 1800 to 1300 K, followed by 10 ps at 1300 K, a 2 ps ramp from 1300 to 900 K, 5 ps at 900 K, a 2 ps ramp from 900 to 600 K, 5 ps at 600 K, and a 2 ps ramp from 600 to 300 K. This was followed by 14 ps at 300 K, and finally by an additional 20 ps plateau at 300 K using the scaled density of Ref.[6]. This final 20 ps segment was used for the vibrational analysis.

The vibrational density of states (VDOS) was calculated from the Fourier transform of the velocity autocorrelation function (VACF). For a given atomic species α, containing N_α atoms, the VACF is defined as

$$C_\alpha(t) = \frac{1}{N_\alpha} \sum_{i \in \alpha} \langle \mathbf{v}_i(t_0) \cdot \mathbf{v}_i(t_0 + t) \rangle_{t_0}\,, \tag{1}$$

where $\mathbf{v}_i(t)$ is the velocity of atom i at time t and $\langle \cdots \rangle_{t_0}$ denotes an average over different time origins along the trajectory. The normalized VACF is given by

$$\widetilde{C}_\alpha(t) = \frac{C_\alpha(t)}{C_\alpha(0)}, \tag{2}$$

and the corresponding VDOS is obtained from

$$g_\alpha(\omega) \propto \int_0^\infty \widetilde{C}_\alpha(t) \cos(\omega t)\, dt, \tag{3}$$

which was evaluated numerically from the discrete Fourier transform of the normalized VACF. The same procedure was applied to obtain the total VDOS by considering all atoms in the simulation cell.

Figure SS5 compares the total VDOS and the element-resolved Si, C, N and H contributions obtained from the FPMD and MACE trajectories. Overall, the MACE potential reproduces the FPMD results with very good agreement over the entire vibrational range. In particular, the positions of the main vibrational bands and the overall spectral distributions associated with each chemical element are consistently reproduced. Some differences in the detailed peak intensities and widths are expected because the spectra are obtained

from finite and independently evolving molecular-dynamics trajectories; nevertheless, the characteristic frequency domains are preserved by the MLIP.

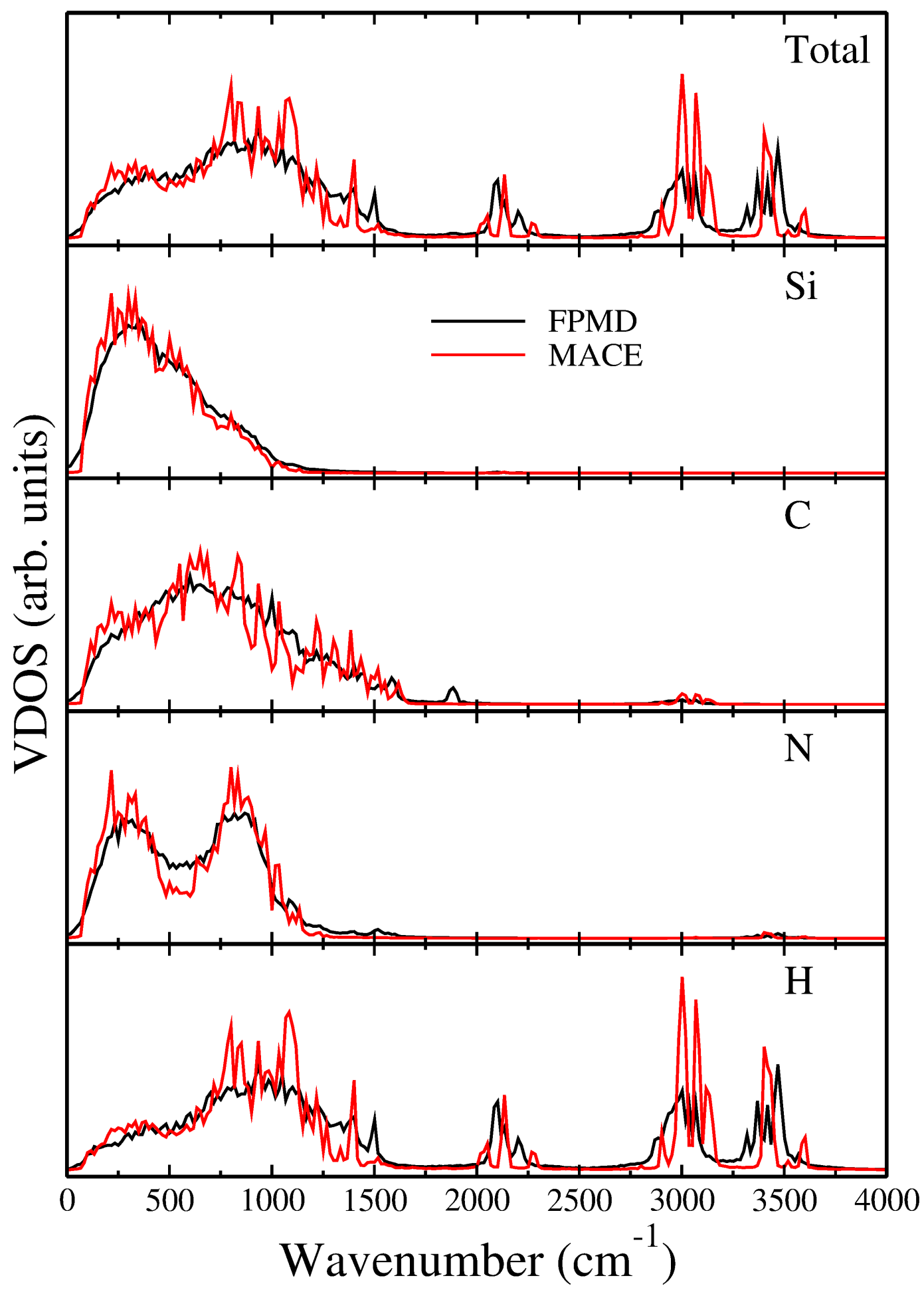

Figure S5: Total and element-resolved vibrational density of states (VDOS) of the 200-atom SiCNH model obtained from FPMD (black) and MACE molecular dynamics (red). The total VDOS and the Si-, C-, N- and H-resolved contributions were calculated from the Fourier transform of the velocity autocorrelation functions evaluated over the final 20 ps of the trajectories at 300 K.

The element-resolved spectra provide further insight into the origin of the different vibrational domains. The Si contribution is confined almost entirely to the low-frequency region below approximately 1200 cm^{-1}. The N contribution is also restricted mainly to the low- and intermediate-frequency range and displays two broad groups of modes, approximately below 500 cm^{-1} and between about 650 and 1200 cm^{-1}. In contrast, the C contribution is distributed over a broader range, extending from low frequencies up to approximately

1600 cm^{-1}. These low- and intermediate-frequency contributions reflect the collective vibrations of the amorphous Si–C–N network. Owing to the strong coupling between atomic motions and the broad distribution of local environments in the amorphous structure, individual features in this region cannot be unambiguously assigned to specific bond vibrations from the element-projected VDOS alone.

The H-resolved VDOS exhibits several well-separated high-frequency features. A band around 2000–2200 cm^{-1} is associated predominantly with Si-H stretching modes, while the intense modes in the approximately 2900–3200 cm^{-1} region originate mainly from C-H stretching vibrations. The highest-frequency features, around 3300–3500 cm^{-1}, are associated with N-H stretching. Lower-frequency H contributions below approximately 1500 cm^{-1} arise from bending, wagging and deformation modes coupled to the Si-C-N network. These assignments are consistent with experimental vibrational bands reported for polysilazane-derived Si-C-N(H)-based materials[12,13] and with previous DFT calculations of vibrational modes in hydrogenated silicon carbonitride systems[14].

Overall, both FPMD and MACE reproduce the same characteristic vibrational domains, including the low-frequency Si-C-N network vibrations and the distinct Si-H, C-H and N-H stretching regions. The close agreement between the total and element-resolved FPMD and MACE VDOS therefore demonstrates that the MLIP reproduces the local curvature of the potential-energy surface governing atomic vibrations. This additional dynamical validation supports the use of the potential for describing the thermally activated bond rearrangements and structural evolution investigated in the large-scale SiCNH simulations.

S7. Initial parameters of the 8000 atoms models

Initial configurations of models SiCNH8000-CNB and SiCNH8000-CL were generated using the ceramicNetworkBuilder program[6] with target bonding and coordination number probabilities given in Tables SS2 and SS3. These parameters allow the construction of an amorphous

network composed of Si, C and N atoms. Hydrogen atoms are inserted afterwards following the target SiCNH composition to fill incomplete atomic local environments as in our previous work[6].

Table S2: Target bonding probabilities used in CeramicNetworkBuilder to generate the initial configurations of SiCNH8000-CNB and SiCNH8000-CL models.

Model	Neighbor type	Central atom type Si	C	N
SiCNH8000-CNB	2	0	0	0
	3	0	20	100
	4	100	80	0
SiCNH8000-CL	2	0	0	0
	3	0	0	100
	4	100	100	0

Table S3: Target coordination number probabilities used in CeramicNetworkBuilder to generate the initial configurations of SiCNH8000-CNB and SiCNH8000-CL models.

Model	Neighbor type	Central atom type Si	C	N
SiCNH8000-CNB	Si	0	80	100
	C	40	20	0
	N	60	0	0
SiCNH8000-CL	Si	0	100	100
	C	25	0	0
	N	75	0	0

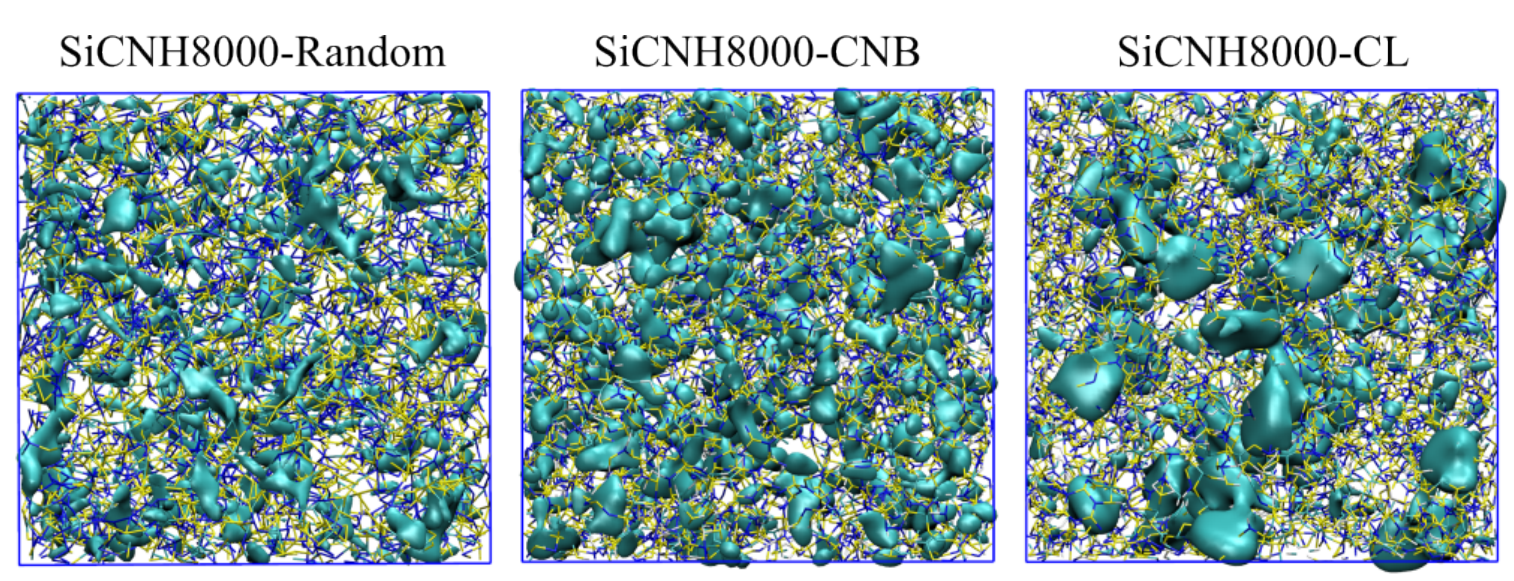

Figure S6: Initial configurations of the SiCNH8000-Random, SiCNH8000-CNB and SiCNH8000-CL models. Turquoise regions indicate carbon-rich domains highlighted in QuickSurf iso-surface representation in VMD[15]. Yellow, blue and white lines represent bonds made by the silicon, nitrogen and hydrogen atoms, respectively.

S8. PDFs scaling and comparison

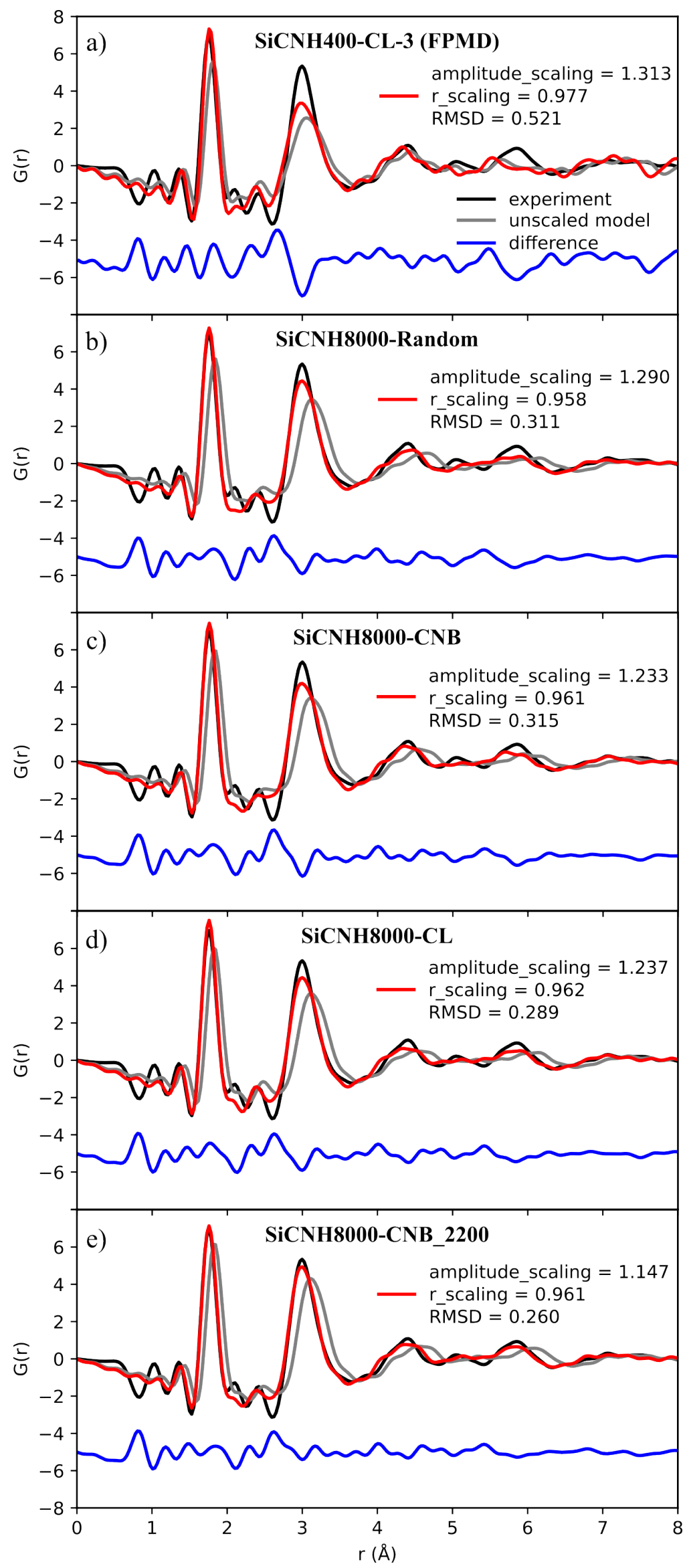

Figure S7: Total X-ray scattering PDF data simulated for all SiCNH PDC models studied in this work, and compared to experimental data (in black). Data in grey are the raw PDF data. Data in red correspond to simulated PDF data wherein an amplitude scaling factor and a r-scaling factor have been optimized to best match to the experiment. The corresponding scaling factor and RMSD are indicated in the legend. Difference between scaled-optimized simulated data and experiment are shown below, in blue.

S9. Atomic local environments in the MLIP 8000-atom models

Table S4: Local atomic environments of the SiCNH8000-Random model. The "Other" sections compile environments whose individual fraction is below 3%.

	Proportion [%]									
Coordination number	l=1		l=2		l=3		l=4		l≥5	
Si	0.1		0.7		8.1		87.1		3.8	
	Other	0.1	Other	0.7	Other	8.1	Si-C_2N_2	24.7	Other	3.8
							Si-CN_3	19.0		
							Si-C_3N	13.9		
							Si-SiC_2N	5.9		
							Si-N_4	5.2		
							Si-SiCN_2	4.0		
							Si-C_4	3.2		
							Other	11.2		
C	0		3.2		37.4		56.9		0	
			Other	3.2	C-Si_2C	7.6	C-Si_3H	22.9		
					C-SiC_2	6.3	C-Si_4	21.1		
					C-Si_3	4.7	C-Si_2H_2	6.4		
					C-C_2N	4.1	Other	9.2		
					C-C_3	3.5				
					C-SiCN	3.3				
					Other	7.9				
N	3.7		7.8		86.5		2.0		0	
	Other	3.7	N-SiC	5.1	N-Si_3	61.0	Other	2.0		
			Other	2.7	N-Si_2H	14.9				
					N-Si_2C	7.0				
					Other	3.6				
H	99.9		0		0		0		0	
	H-C	66.2								
	H-N	22.6								
	H-Si	11.0								
	H-H	0.1								

Table S5: Local atomic environments of the SiCNH8000-CNB model. The "Other" sections compile environments whose individual fraction is below 3%.

	Proportion [%]									
Coordination number	l=1		l=2		l=3		l=4		l≥5	
Si	0.1		0.6		5.7		88.8		4.7	
	Other	0.1	Other	0.6	Other	5.7	$Si\text{-}C_2N_2$	27.1	Other	4.7
							$Si\text{-}CN_3$	21.2		
							$Si\text{-}C_3N$	14.3		
							$Si\text{-}N_4$	7.6		
							$Si\text{-}SiC_2N$	3.6		
							$Si\text{-}C_4$	3.0		
							Other	12.0		
C	0		2.1		42.0		56.0		0	
			Other	2.1	$C\text{-}SiC_2$	12.5	$C\text{-}Si_3H$	21.1		
					$C\text{-}Si_2C$	10.4	$C\text{-}Si_4$	17.9		
					$C\text{-}C_3$	6.2	$C\text{-}Si_2H_2$	6.2		
					$C\text{-}Si_3$	3.4	$C\text{-}Si_2CH$	3.4		
					Other	9.5	$C\text{-}Si_3C$	3.2		
							Other	4.2		
N	2.3		2.1		93.5		2.2		0	
	Other	2.3	Other	2.1	$N\text{-}Si_3$	71.9	Other	2.2		
					$N\text{-}Si_2H$	17.0				
					$N\text{-}Si_2C$	3.3				
					Other	1.3				
H	100.0		0		0		0		0	
	H-C	63.5								
	H-N	23.9								
	H-Si	12.1								
	H-H	0.5								

Table S6: Local atomic environments of the SiCNH8000-CL model. The "Other" sections compile environments whose individual fraction is below 3%.

Coordination number	Proportion [%] l=1	l=2	l=3	l=4	l≥5
Si	0	0.6	6.8	88.6	4.1
		Other 0.6	Other 6.8	$Si\text{-}CN_3$ 23.3	Other 4.1
				$Si\text{-}C_2N_2$ 20.5	
				$Si\text{-}C_3N$ 11.4	
				$Si\text{-}N_4$ 9.5	
				$Si\text{-}SiCN_2$ 3.5	
				$Si\text{-}SiC_2N$ 3.4	
				$Si\text{-}C_4$ 3.1	
				$Si\text{-}CN_2H$ 3.0	
				Other 10.9	
C	0	0.5	47.0	52.5	0
		Other 0.5	$C\text{-}SiC_2$ 16.2	$C\text{-}Si_3H$ 21.1	
			$C\text{-}C_3$ 16.1	$C\text{-}Si_4$ 18.0	
			$C\text{-}Si_2C$ 4.1	$C\text{-}Si_2H_2$ 5.9	
			$C\text{-}Si_3$ 4.0	Other 7.5	
			$C\text{-}C_2H$ 3.0		
			Other 3.6		
N	0.2	2.2	95.3	2.4	0
	Other 0.2	Other 2.2	$N\text{-}Si_3$ 75.6	Other 2.4	
			$N\text{-}Si_2H$ 16.9		
			Other 2.8		
H	100.0	0	0	0	0
	H-C 58.8				
	H-N 24.4				
	H-Si 16.4				
	H-H 0.4				

Table S7: Local atomic environments of the SiCNH8000-CNB_2200 model. The "Other" sections compile environments whose individual fraction is below 3%.

	Proportion [%]				
Coordination number	l=1	l=2	l=3	l=4	l≥5
Si	0	0.4	5.5	89.8	4.2
		Other 0.4	Other 5.5	$Si\text{-}C_2N_2$ 27.9	Other 4.2
				$Si\text{-}CN_3$ 23.3	
				$Si\text{-}C_3N$ 14.0	
				$Si\text{-}N_4$ 8.3	
				$Si\text{-}SiC_2N$ 3.0	
				Other 13.3	
C	0	0.3	41.7	58.0	0
		Other 0.3	$C\text{-}C_3$ 15.0	$C\text{-}Si_4$ 24.2	
			$C\text{-}SiC_2$ 12.3	$C\text{-}Si_3H$ 20.8	
			$C\text{-}Si_3$ 4.9	$C\text{-}Si_2H_2$ 7.5	
			$C\text{-}C_2H$ 3.9	Other 5.4	
			Other 5.7		
N	0.2	2.2	93.9	3.7	0
	Other 0.2	Other 2.2	$N\text{-}Si_3$ 76.6	Other 3.7	
			$N\text{-}Si_2H$ 13.8		
			Other 3.5		
H	100.0	0	0	0	0
	H-C 65.3				
	H-N 21.6				
	H-Si 11.9				
	H-H 1.2				

S10. Visualization of the progressive evolution of carbon domain organization

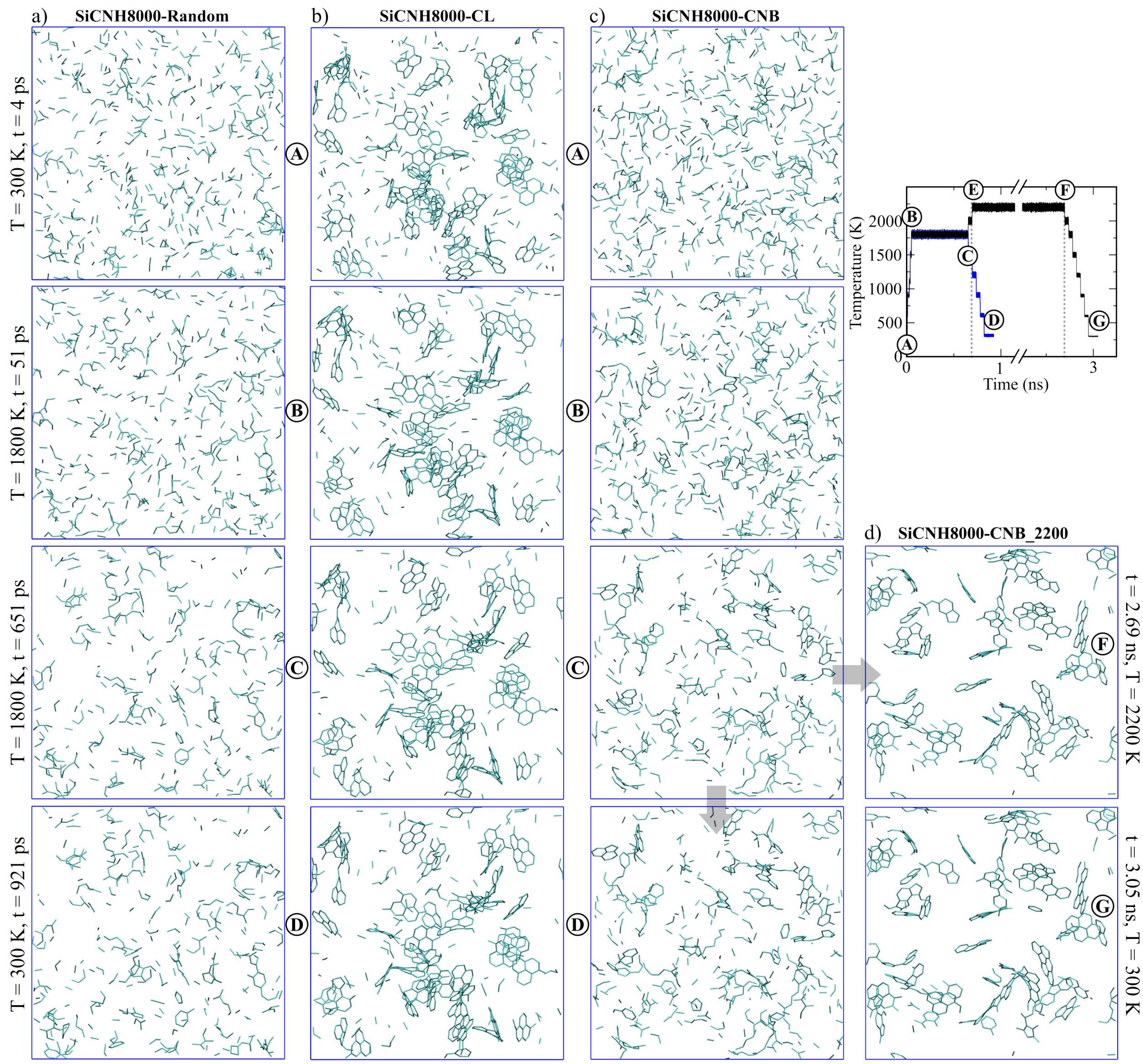

Figure S8: Configuration of C-C bonds in the (a) SiCNH8000-Random, (b) SiCNH8000-CL, (c) SiCNH8000-CNB, and (d) SiCNH8000-CNB_2200 models at different stages (A-D for the former three models and F-G for the latter, heated up to 2200 K) of the thermal cycles, shown for convenience on the top right side of the figure. The progressive evolution of carbon domain organization is clearly visible, with significant differences in the initial configurations leading to distinct pathways of carbon structuration.

S11. Evolution of the number of carbon rings

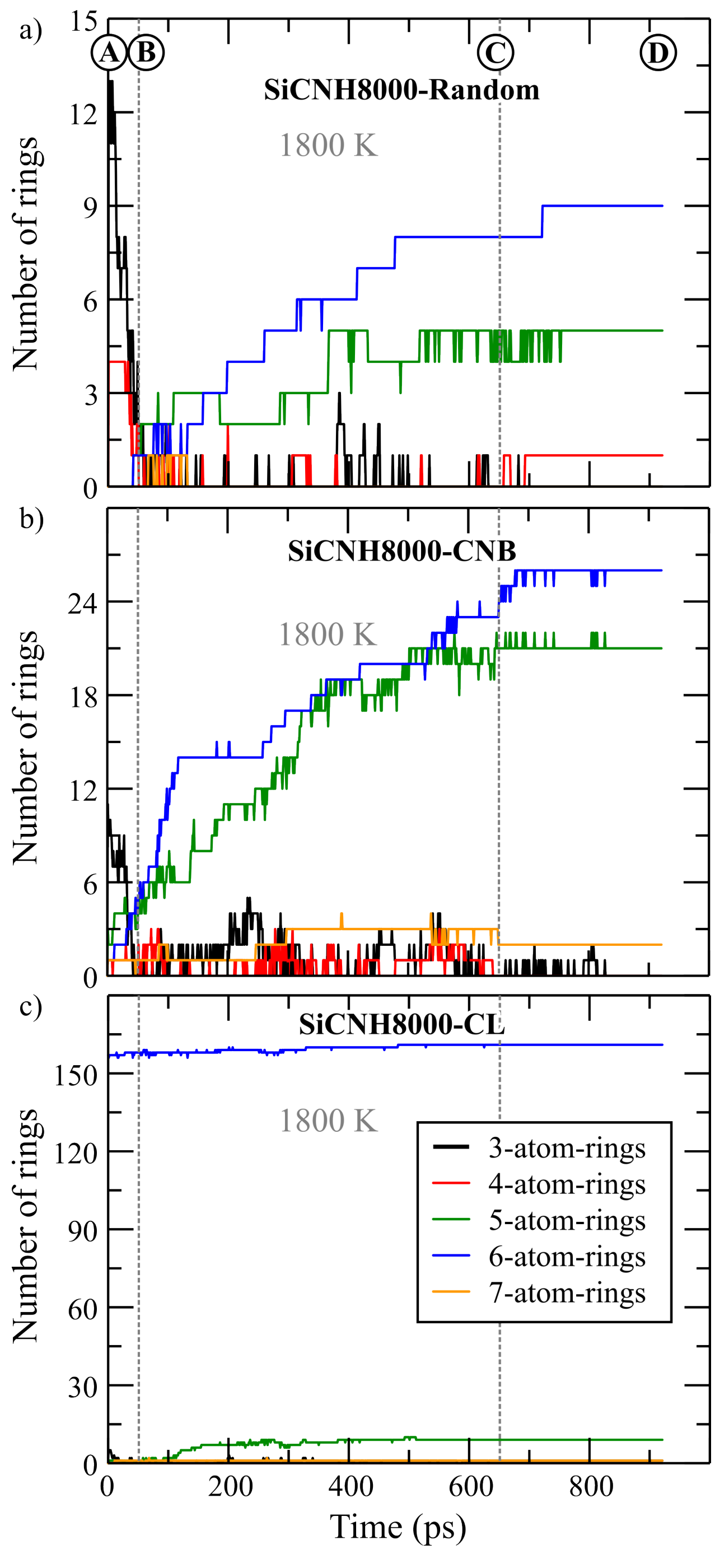

Figure S9: Evolution of the number of carbon rings with different sizes during the thermal cycle. The vertical dashed lines indicate the 1800 K temperature plateau, marking a period of accelerated ring formation and transformation. Circled A-D capital letters indicate the timing of configurations shown in Figure SS8.

S12. The C–C–C bond-angle distributions

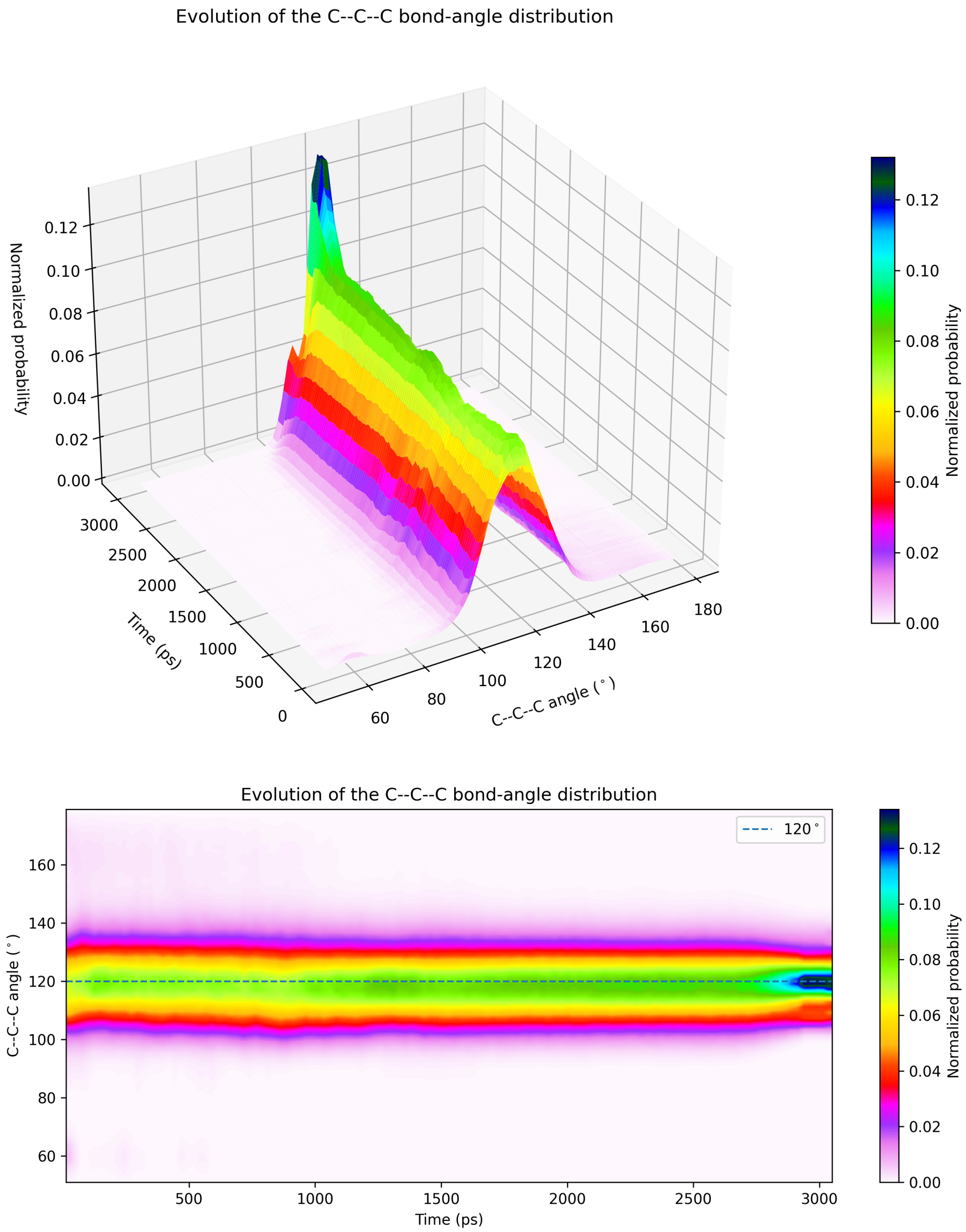

Figure S10: C–C–C bond-angle distributions (BADs) calculated along the complete thermal cycle of the SiCNH8000-CNB_2200 model. Top: 3D representation of the BAD averaged over successive 25 ps time windows. Bottom: projection of the same data as an angle–time heat map. In each 25 ps window, all C–C–C angles were accumulated using a C–C cutoff of 1.90 Å and the resulting histogram was normalized. The dashed line marks 120°, characteristic of trigonal-planar carbon geometry.

To complement the topological analysis of the carbon phase, we calculated the C–

C–C bond-angle distribution (BAD) along the full molecular-dynamics trajectory of the SiCNH8000-CNB_2200 model. This analysis provides a direct geometric characterization of the carbon environments associated with the evolving free-carbon domains.

The trajectory was saved every 200 fs, and periodic boundary conditions were applied. C–C bonds were identified using a distance cutoff of 1.90 Å. For each carbon atom having at least two C nearest neighbors, all distinct C–C–C angles centered on that atom were calculated. The set of angles obtained in this way for a given trajectory segment defines the corresponding C–C–C BAD.

To improve the statistical quality of the distributions and to follow the evolution of the carbon phase during the full thermal treatment, the angles were accumulated over successive 25 ps time windows. For each time window, the angular histogram was constructed using 2° bins between 50° and 180° and normalized by the total number of angles accumulated in that window, allowing the resulting BADs to be directly compared throughout the full thermal cycle.

Figure SS10 shows the 3D representation of the C–C–C BAD (top panel) together with its 2D heat-map projection (bottom panel). Over the full thermal cycle, the distribution extends mainly between approximately 105° and 135°, with a maximum near 120° indicating that the carbon motifs largely adopt planar or near-planar geometries. During the 2200 K plateau, from 651 to 2651 ps, the relative intensity around 120° progressively increases while the temperature remains constant, revealing a structural reorganization of the carbon phase toward more trigonal-planar local geometries. During the subsequent cooling stage, the distribution around 120° becomes noticeably sharper, which is consistent with an additional reduction of thermal broadening.

This geometric evolution is fully consistent with the connectivity-based analysis reported in the main text. In parallel with the decrease of carbon dimers and chains and the growth of ring-containing carbon sheets, the BAD shows an increasing predominance of angular populations characteristic of trigonal-planar carbon arrangements. Consequently, one can

infer that the developing free-carbon domains adopt progressively more ordered sp^2-like local environments.

References

(1) Kühne, T. D. et al. CP2K: An electronic structure and molecular dynamics software package - Quickstep: Efficient and accurate electronic structure calculations. *J. Chem. Phys.* **2020**, *152*, 194103.

(2) Nosé, S. A molecular dynamics method for simulations in the canonical ensemble. *Mol. Phys.* **1984**, *52*, 255–268.

(3) Nosé, S. A unified formulation of the constant temperature molecular dynamics methods. *J. Chem. Phys.* **1984**, *81*, 511–519.

(4) Hoover, W. G. Canonical dynamics: Equilibrium phase-space distributions. *Phys. Rev. A* **1985**, *31*, 1695–1697.

(5) Martyna, G. J.; Klein, M. L.; Tuckerman, M. Nos'e–Hoover chains: The canonical ensemble via continuous dynamics. *J. Chem. Phys.* **1992**, *97*, 2635–2643.

(6) Mortier, F.; Cadars, S.; Miled., M. B.; Masson, O.; Ori, G.; Boero, M.; Wang, Y.; Bernard, S.; Bouzid, A. First-principles modeling of polysilazane derived SiCNH ceramics: insights into the organization of the free carbon phase. *Phys. Chem. Chem. Phys.* **2026**,

(7) Kühne, T. D.; Krack, M.; Mohamed, F. R.; Parrinello, M. Efficient and Accurate Car-Parrinello-like Approach to Born-Oppenheimer Molecular Dynamics. *Phys. Rev. Lett.* **2007**, *98*, 066401.

(8) Kühne, T. D. Second generation Car–Parrinello molecular dynamics. *WIREs Computational Molecular Science* **2014**, *4*, 391–406.

(9) Perdew, J. P.; Burke, K.; Ernzerhof, M. Generalized Gradient Approximation Made Simple. *Phys. Rev. Lett.* **1996**, *77*, 3865–3868.

(10) Grimme, S.; Antony, J.; Ehrlich, S.; Krieg, H. A consistent and accurate ab initio parametrization of density functional dispersion correction (DFT-D) for the 94 elements H-Pu. *J. Chem. Phys.* **2010**, *132*, 154104.

(11) Valle, M.; Oganov, A. R. Crystal fingerprint space – a novel paradigm for studying crystal-structure sets. *Acta Crystallogr. Sect. A* **2010**, *66*, 507–517.

(12) Morais Ferreira, R. K.; Ben Miled, M.; Nishihora, R. K.; Christophe, N.; Carles, P.; Motz, G.; Bouzid, A.; Machado, R.; Masson, O.; Iwamoto, Y.; Célérier, S.; Habrioux, A.; Bernard, S. Low temperature in situ immobilization of nanoscale fcc and hcp polymorphic nickel particles in polymer-derived Si–C–O–N(H) to promote electrocatalytic water oxidation in alkaline media. *Nanoscale Adv.* **2023**, *5*, 701–710.

(13) Ben Miled, M.; Fradin, M.; Benbakoura, N.; Mazière, L.; Rousseau, J.; Bouzid, A.; Carles, P.; Iwamoto, Y.; Masson, O.; Habrioux, A.; Bernard, S. Encapsulating Nickel-Iron Alloy Nanoparticles in a Polysilazane-Derived Micro-/Mesoporous Si-C-O-N-Based Support to Stimulate Superior OER Activity. *ChemSusChem* **2024**, *n/a*, e202400561.

(14) Coustel, R.; Haacké, M.; Rouessac, V.; André, E.; Roualdes, S.; Julbe, A. Vibrational frequencies of hydrogenated silicon carbonitride: A DFT study. *Surface and Coatings Technology* **2017**, *325*, 437–444.

(15) Humphrey, W.; Dalke, A.; Schulten, K. VMD: visual molecular dynamics. *J. Mol. Graph.* **1996**, *14*, 33–38.
